# Assembled molecular face-rotating polyhedra to transfer chirality from two to three dimensions

**DOI:** 10.1038/ncomms12469

**Published:** 2016-08-24

**Authors:** Xinchang Wang, Yu Wang, Huayan Yang, Hongxun Fang, Ruixue Chen, Yibin Sun, Nanfeng Zheng, Kai Tan, Xin Lu, Zhongqun Tian, Xiaoyu Cao

**Affiliations:** 1 State Key Laboratory of Physical Chemistry of Solid Surfaces and College of Chemistry and Chemical Engineering, Xiamen University, Xiamen 361005, China; 2 Collaborative Innovation Centre of Chemistry for Energy Materials, Xiamen University, Xiamen 361005, China

## Abstract

In nature, protein subunits on the capsids of many icosahedral viruses form rotational patterns, and mathematicians also incorporate asymmetric patterns into faces of polyhedra. Chemists have constructed molecular polyhedra with vacant or highly symmetric faces, but very little is known about constructing polyhedra with asymmetric faces. Here we report a strategy to embellish a *C*_3h_ truxene unit with rotational patterns into the faces of an octahedron, forming chiral octahedra that exhibit the largest molar ellipticity ever reported, to the best of our knowledge. The directionalities of the facial rotations can be controlled by vertices to achieve identical rotational directionality on each face, resembling the homo-directionality of virus capsids. Investigations of the kinetics and mechanism reveal that non-covalent interaction among the faces is essential to the facial homo-directionality.

## Abstract (web summary)

Protein subunits on the capsids of icosahedral viruses can form patterns with rotational symmetry, which are difficult to recreate in the laboratory. Here the authors report a strategy to construct 3D chiral polyhedra with rotational faces from 2D chiral truxene-based units through dynamic covalent chemistry.

## Graphical abstract

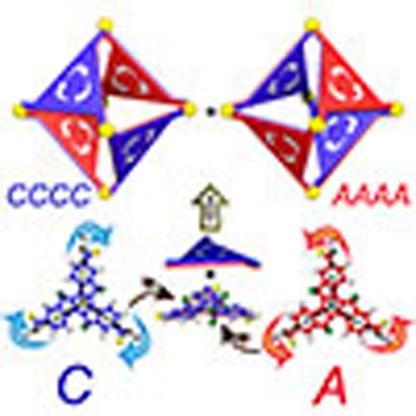

Motifs in architecture, mathematics and nature have inspired chemists to create their molecular counterparts^1^,^2^,^3^,^4^,^5^,^6^,^7^,^8^,^9^. Buckminsterfullerene (C_60_)^1^, for instance, structurally resembles the geodesic dome by Buckminster Fuller. Fuller also conceived of face-rotating polyhedra (FRP)^10^, wherein each face of a polyhedron rotates around its central axis, transferring the two-dimensional (2D) chirality of the faces into the 3D chirality of the polyhedron (Fig. 1a). In mathematics, chirality transfer in FRP is highlighted as an analogous strategy to construct polyhedral dice^11^. In life sciences, some virus capsids can be regarded as FRP^12^,^13^,^14^,^15^, in which each face of the icosahedral cricket paralysis virus capsid contains three quasi-equivalent subunits that form identical rotational directionality (Fig. 1b)^14^. The transfer of 2D chirality of the building block to 3D chirality in FRP through self-assembly can be regarded as a type of emergent property^16^,^17^,^18^.

Inspired by mathematics^19^ and the structures of virus capsids^20^, chemists have synthesized molecular polyhedra with various sizes and shapes through self-assembly. Most of these polyhedra are highly symmetric and achiral^20^,^21^,^22^,^23^,^24^,^25^, and chiral molecular polyhedra with lower symmetry have attracted more and more attention because they increase the complexity of motifs and may bring more sophisticated functions^26^,^27^,^28^,^29^,^30^,^31^. Generally, the construction of chiral molecular polyhedra follows two strategies: to use predetermined 3D chiral building blocks as the edges^30^,^32^, vertices^33^,^34^ or faces^35^,^36^ of polyhedra to transfer 3D chirality of the components to the 3D chirality of the chiral structures, or to use achiral components to generate chiral metal-organic polyhedra^37^,^38^,^39^,^40^,^41^,^42^ during a self-assembly process. The latter approach relates to symmetry breaking, and investigation of the process of such complex molecular self-assembly may help to elucidate the evolution of matter towards complexity^43^. However, metal-organic polyhedra remain the dominant type of chiral molecular polyhedra assembled from achiral components, and the resolution of the mixed racemic structures as well as scrutiny of the intermediates and the formation mechanism are challenging.

Here we report a strategy to assemble a *C*_3h_-symmetric building block into purely organic FRP (Fig. 1c) through dynamic covalent chemistry^44^,^45^,^46^. The building block loses its σ_h_ mirror symmetry in the polyhedra and brings either a clockwise (*C*) or an anticlockwise (*A*) rotation around the centre of each face, thus generating a series of FRP stereoisomers. Our thermodynamic polyhedra exhibit homo-directionality, which resembles the identical rotational directionality of each facial unit in some virus capsids. We also investigated the formation mechanisms of the FRP from rotational facial units based on their high spectral sensitivities. The non-covalent interaction among faces has been suggested to be the primary reason for the facial homo-directionality.

## Results

### Synthesis of FRP 1 with achiral vertices

**Figure 1:**
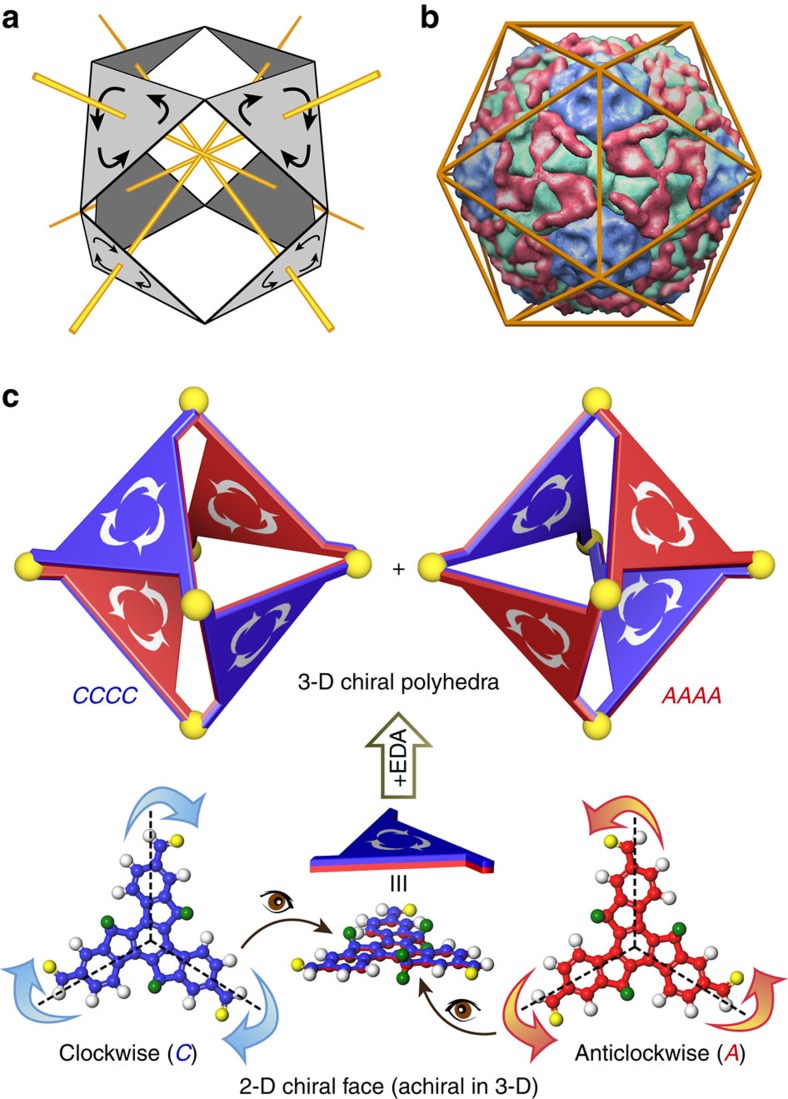
3D chiral polyhedra originated from 2D chiral facial units. (**a**) Synergetic interactions between the rotating triangular faces in a polyhedron proposed by Buckminster Fuller. (**b**) All subunits of icosahedral cricket paralysis virus have identical rotational directionality. (**c**) Truxene building block shown as a ball-and-stick model (red and blue, carbon; white, hydrogen; yellow, oxygen; green, butyl group); its rotation patterns are considered to be either clockwise (*C*) or anticlockwise (*A*) when viewed vertical to the aromatic plane. By reacting with ethylene diamine to form an octahedron, the truxene fragment loses its mirror symmetry and becomes a rotational face.

The facial building block **TR** (Fig. 1c and Supplementary Fig. 1) consists of a rigid planar 10,15-dihydro-5*H*-diindeno[1,2-*a*;10,20-*c*]fluorine (truxene) core^47^,^48^, six alkyl chains at central positions to increase solubility and prevent aggregation, and three carboxaldehyde groups at peripheral positions for reversible imine formation. This building block was readily synthesised from truxene in three steps with high yields (Supplementary Fig. 2).

**Figure 2:**
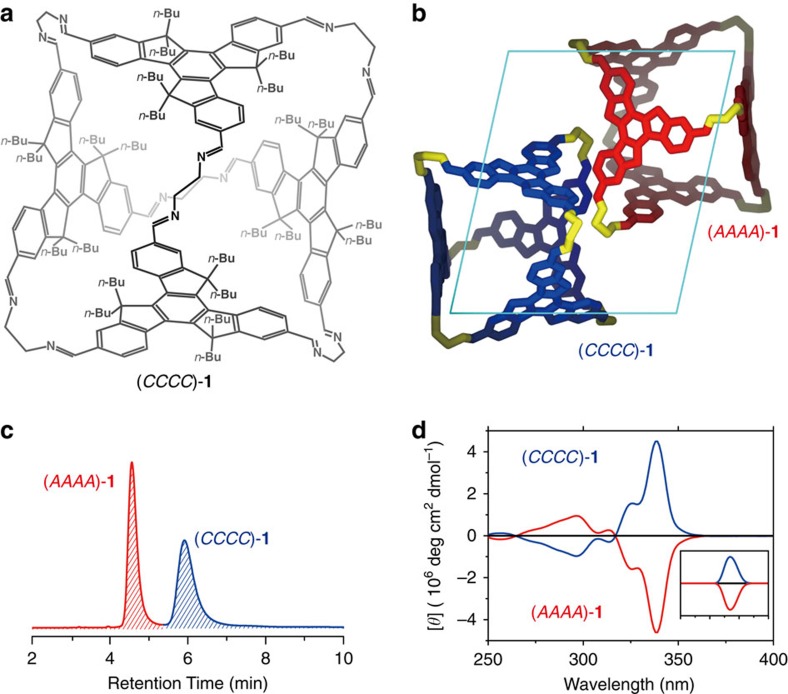
Molecular structure and characterisation of octahedra 1. (**a**) Molecular structure of the octahedron (*CCCC*)-**1**. (**b**) Single-crystal structure of the co-crystal of (*CCCC*)-**1** and (*AAAA*)-**1** (hydrogen atoms and butyl groups are omitted for clarity). (**c**) Chiral HPLC spectrum contains two peaks corresponding to (*AAAA*)-**1** and (*CCCC*)-**1** in a 1:1 ratio. The absorbance for all octahedra was recorded at 325 nm. (**d**) Experimental and (ZINDO/S)-predicted (the insert) CD spectra of (*AAAA*)-**1** (red), (*CCCC*)-**1** (blue), and the unseparated racemic mixture (black) in hexane.

The truxene building block (**TR**) (4 eq) and ethylene diamine (EDA) (6 eq) were reacted in toluene with catalytic amounts of trifluoroacetic acid (TFA) at room temperature, affording TR_4_EDA_6_ octahedron **1** in high yield. The mass spectrum of **1** confirmed that it was formed exclusively in solution and exhibited a molecular weight (M.W.) of 3196.29 daltons (calculated M.W.: 3196.31), corresponding to a [4+6] composition (Fig. 2a).

### Single-crystal characterization of FRP 1

Single crystals were grown by diffusing hexane into the toluene solution of **1**. Single-crystal X-ray diffraction analysis revealed that **1** has a tetra-capped octahedral structure^21^,^33^, in which four truxene units occupy four faces linked by six diamine units in the vertices (Fig. 2b). Truxene is achiral in three dimensions because of the presence of a mirror symmetry, and chiral in two dimensions because the three sp^3^ carbons of the truxene core form a rotating pattern along the *C*_3_ axis in either a clockwise or an anticlockwise direction (Fig. 1c), similar to the pattern in some virus capsids. Therefore, the truxene fragment loses its mirror symmetry in a polyhedron and becomes a rotational face, resulting in five possible stereoisomers for **1**; these configurations are named after the directionalities of the exterior faces as *CCCC* and *AAAA* (homo-directional with *T* symmetry), *CCCA* and *CAAA* (hetero-directional with *C*_*3*_ symmetry) and *CCAA* (hetero-directional with *S*_*4*_ symmetry) (Supplementary Fig. 1). We measured more than five single crystals of **1**, and found all of them were the co-crystallization of only the *T*-symmetric enantiomers (i.e., (*CCCC*)-**1** and (*AAAA*)-**1**) packed in the *P*-1 triclinic space group (Fig. 2b). No other diastereomers or self-sorting of the two enantiomers into two enantiopure crystals was observed. All imine bonds are *E* conformers, locating in the conjugated plane and rotating in the same direction as the sp^3^ carbons of the truxene core. The butyl groups compacted inside the octahedra distort the terminal carbons, whereas the butyl groups that stretch outside remain linear.

### NMR characterization of FRP 1

**Figure 3:**
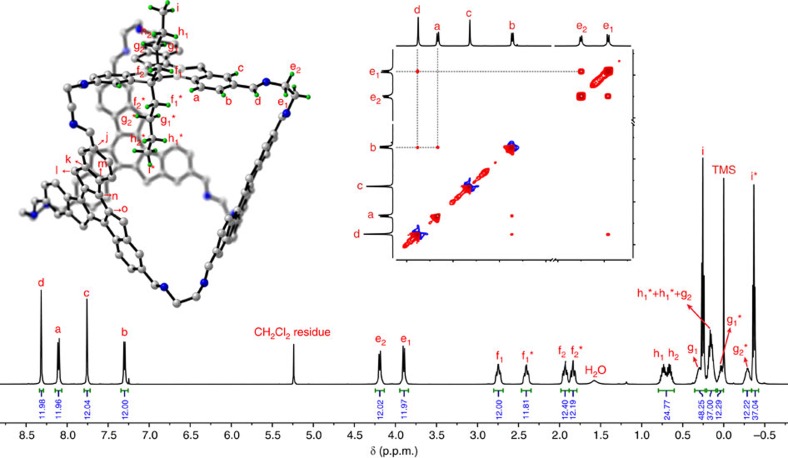
^1^H NMR and NOE spectra of octahedron 1. ^1^H NMR spectrum was measured in dichloromethane and fully rationalised by matching the crystal structure and 2D NMR spectra. The inserted NOE spectrum exhibits the crosspeaks of H^d^–H^b^ and H^d^–H^e1^, suggesting H^d^ is spatially close to H^b^ and H^e1^ in solution, as presented in the molecular structure (only one butyl chain is shown for clarity).

The nuclear magnetic resonance (NMR) spectra of **1** (Supplementary Figs 3–9) are consistent with its single-crystal structure. The ^1^H NMR spectrum (Fig. 3) reveals only a single set of peaks for all twelve sets of protons on the truxene backbones (H^a^, H^b^, and H^c^) and imines (H^d^), suggesting that these protons are individually interchangeable through twelve symmetry operations^49^. Therefore, only the *T*-symmetric stereoisomers of **1** exist in solution. All butyl protons inside the octahedron are more shielded from the three neighbouring truxene units than those outside. The 2D NMR spectra allowed for the assignment of each proton and carbon of octahedron **1**. In addition, the nuclear Overhauser effect (NOE) crosspeak between imine protons H^d^ and H^b^ (instead of H^c^) suggests that all imine bonds rotate in the same direction as the sp^3^ carbons of the truxene core in solution, and the crosspeak between H^d^ and H^e1^ reflects the shape-persistency and rigidity of the octahedron **1** in solution (Fig. 3, insert).

### Chiral-HPLC and CD characterizations of FRP 1

Further application of chiral high-performance liquid chromatography (HPLC) and circular dichroism (CD) spectroscopy confirmed that, among all possible stereoisomers, only (*CCCC*)-**1** and (*AAAA*)-**1** exist in a 1:1 ratio both in crystal and in solution (Fig. 2c). The solution of crude **1** gives no detectable CD signal. In contrast, the CD signal of the separated octahedron eluting at 4.6 min gives a strong negative signal in the truxene absorption region (340 nm), whereas the fraction at 5.9 min provides a mirror-like positive signal (Fig. 2d). The CD signals of both separated octahedra have a molar ellipticity of 4.6 × 10^6^ deg cm^2^ dmol^−1^, which, to our knowledge, is the largest molar ellipticity ever reported^50^ and is substantially larger than that of helicene (*ca.* 3.5 × 10^5^ deg cm^2^ dmol^−1^)^51^. We attribute this large molar ellipticity to the high molar absorptivity of truxene and to the rigid geometry of the octahedra. The CD spectrum of the octahedron at 4.6 min matches the (ZINDO/S)-predicted CD spectrum of (*AAAA*)-**1**, and the CD spectrum at 5.9 min corresponds to (*CCCC*)-**1** (Fig. 2d, insert).

### Synthesis and characterizations of FRP 2 with chiral vertices

After achieving homo-directionality of each face of the molecular polyhedra, we attempted the enantioselective assembly of FRP, similar to the assembly observed in nature for some virus capsids^12^,^13^,^14^,^15^. Inspection of the single-crystal structure of **1** revealed that all EDA vertices of (*AAAA*)-**1** are in a gauche conformation, and the two amino groups have a torsion angle of *ca.* −60°, which is the same torsion angle between the amino groups in (*R*,*R*)-diaminocyclohexane (CHDA) (Supplementary Fig. 10). Similarly, the amino groups in the vertices of (*CCCC*)-**1** and (*S*,*S*)-CHDA exhibit a torsion angle of *ca.* 60°. Therefore, we envisioned that the conformational preorganisation of diamines in (*R*,*R*)-CHDA or (*S*,*S*)-CHDA would dominate the facial directionality of the octahedra.

**Figure 4:**
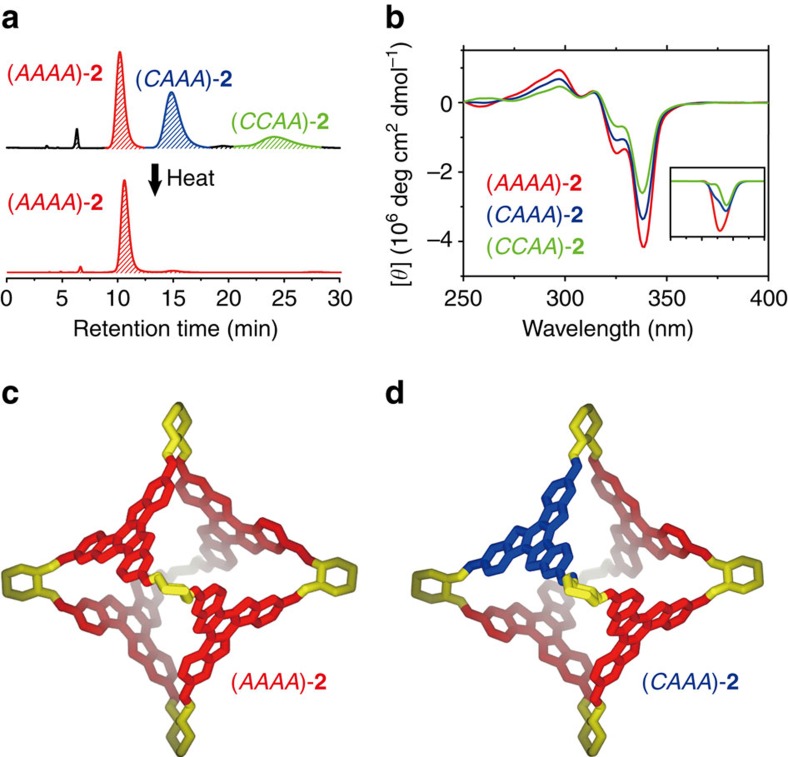
Characterisation of octahedra 2. (**a**) Chiral HPLC spectra of the kinetic products of **2** synthesised at 25 °C (top) revealing three diastereomers (i.e., (*AAAA*)-**2**, (*CAAA*)-**2** and (*CCAA*)-**2**) that changed into the thermodynamic product (*AAAA*)-**2** when heated at 110 °C for 48 h (bottom). (**b**) Experimental and (ZINDO/S)-predicted (the insert) CD spectra of (*AAAA*)-**2** (red), (*CAAA*)-**2** (blue) and (*CCAA*)-**2** (green) in hexane. (**c**,**d**) Single-crystal structures of the thermodynamic (*AAAA*)-**2** (**c**) and kinetically stable (*CAAA*)-**2** (**d**) (hydrogen atoms and butyl groups are omitted for clarity).

Under the same conditions as those used to prepare **1**, (*R*,*R*)-CHDA and **TR** were reacted at room temperature for 24 h. The mass spectrum of the product suggested the exclusive formation of TR_4_CHDA_6_ with a M.W. of 3520.52 (calculated M.W.: 3520.59), corresponding to octahedron **2**. However, the ^1^H NMR spectrum exhibited eleven signals for the imine protons (H^d^), indicating a mixture of diastereomeric octahedra with all three symmetries, *T*, *C*_*3*_ and *C*_*2*_ (Supplementary Fig. 11a). HPLC analysis revealed a distribution of three diastereomers, which eluted at 10.3 min (42%), 14.9 min (42%) and 24.1 min (14%) (Fig. 4a). We isolated each diastereomer and determined its symmetry through the number of imine signals in the NMR spectra. The *T*-, *C*_*3*_-, and *C*_*2*_-symmetric diastereomers that eluted at 10.3, 14.9, and 24.1 min gave rise to a single H^d^ signal, four H^d^ signals and six H^d^ signals, respectively (Supplementary Figs 11–14). The *C*_*2*_-symmetric diastereomer (*CCAA*)-**2** partially converted into other diastereomers after 24 h, thus suggesting that it is a kinetic product. To convert all kinetic products to their corresponding thermodynamic products, we heated **2** at 110 °C for 48 h after stirring at room temperature for 24 h. HPLC and NMR analyses indicated the near quantitative transformation to the *T*-symmetric diastereomer, which eluted at 11.2 min (Fig. 4a and Supplementary Fig. 11b).

On the basis of single-crystal X-ray diffraction analysis, we confirmed the *T*- and *C*_*3*_-symmetric diastereomers to be (*AAAA*)-**2** and (*CAAA*)-**2** octahedra, respectively (Fig. 4c,d). The chirality of the octahedra was inherited from that of the (*R*,*R*)-CHDA vertices. As in (*AAAA*)-**1**, all imine bonds in (*AAAA*)-**2** and the *A* faces of (*CAAA*)-**2** rotate towards the sp^3^ carbons of truxene, whereas the *A* face of (*AAAA*)-**2** rotates against the sp^3^ carbons to match the gauche conformation of the diamine vertex. This response in the imine arrangements to the directional change of the truxene faces is rare, presumably due to the rigidity in the diamine vertices, truxene skeletons and imine junctions.

The structural change in these diastereomers also leads to significantly different spectroscopic results. The CD spectra of (*AAAA)-***2**, (*CAAA)-***2** and (*CCAA*)*-***2** are similar to but sequentially weaker than that of (*AAAA*)-**1**, consistent with the (ZINDO/S)-predicted CD spectra (Fig. 4b, insert). This trend can be rationalized by the decrease in the alternate angles of the truxene cores (Supplementary Fig. 15).

As expected, the enantiomeric vertex of (*S*,*S*)-CHDA led to enantiomeric formation of the thermodynamic octahedron (*CCCC*)-**3** through the (*CCCA*)-**3** and (*CCAA*)-**3** kinetic octahedra (Supplementary Fig. 16).

### Kinetics of the synthesis of FRP 2

**Figure 5:**
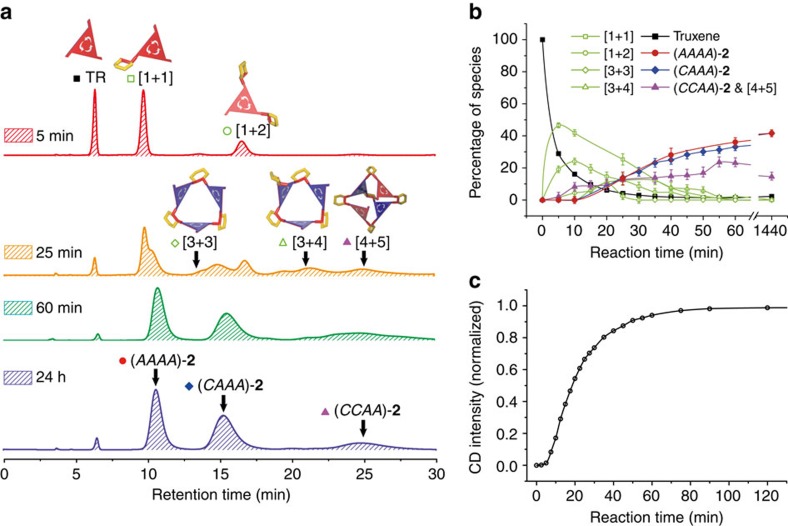
Time-dependent characterisation of the synthesis of octahedron 2. (**a**) Selected HPLC spectra at certain time intervals. The components of the intermediates were determined by mass spectrometry. (**b**) Kinetic profile of the synthesis of octahedron **2** (1.6 mM at 298 K, absorption monitored at 325 nm), showing the consumption of truxene and the formation of three octahedral diastereomers. The formation and disappearance of the intermediates with the [1+1], [1+2], [3+3], [3+4] and [4+5] components can also be tracked. The percentages were evaluated from the HPLC peak areas. (**c**) Time-dependent CD intensities at 340 nm normalised by the intensity at 24 h.

The formation of octahedron **2** at 298 K constitutes a kinetically controlled dynamic combinatorial library^6^,^46^. Therefore, we monitored the library, which contained 1.6 mM **TR**, by time-dependent chiral-HPLC-MS, UV–vis and CD to investigate the possible mechanistic pathways^6^,^52^,^53^ (Fig. 5). In 5 min, most of the **TR** reacted with diamine to form the [1+1] and [1+2] intermediates, which gave rise to a negligible CD signal. In 25 min, the reaction generated more [3+3], [3+4], and [4+5] intermediates, which exhibited a strong CD signal. All intermediates disappeared at 60 min except for the [4+5] intermediate, which gradually transformed into the [4+6] octahedra after 24 h. At room temperature, the formation and cleavage of an imine bond is fast but the transformation from the kinetic (*CAAA*)-**2** and (*CCAA*)-**2** to the thermodynamic (*AAAA*)-**2** was extremely slow. Therefore, we postulated that the cyclohexyl group of CHDA shields the imine bonds in the octahedral ‘testudo formation' in a cooperative manner.

### Reasons for the facial homo-directionality in FRP 1 and 2

To shed light on the homo-directionality of the *CCCC* or *AAAA* configuration of all thermodynamic products of octahedra **1**, **2** and **3**, we calculated the free energies of different stereoisomers by the Compass II force field. The (*CCCC*)-**1** and (*AAAA*)-**1** enantiomers had the lowest energies among all stereoisomers of **1**, whereas the enantiomeric production of (*AAAA*)-**2** and (*CCCC*)-**3** can be rationalized by their relative energies being the lowest among all stereoisomers of **2** and **3** (Table 1). In addition, the energy differences among the diastereomers were primarily due to non-covalent contacts and repulsion forces of the butyl groups inside the octahedra. For illustration, the energy of (*AAAA*)-**1** was calculated to be 12.2 kcal mol^−1^ lower than that of (*CAAA*)-**1**, in which 9.7 kcal mol^−1^ was attributed to the difference in van de Waals interaction. This phenomenon is correlated to the crowded interior of the octahedra in the crystal structures, as well as in the simulated structures (Supplementary Fig. 17). Therefore, we speculate that the non-covalent repulsion forces among the interior butyl groups are essential for directing the homo-directional facial rotations of the thermodynamic polyhedra.

## Discussion

We introduced 2D chiral truxene building block to generate a series of 3D chiral polyhedra with rotational faces. The homo-directionality in our thermodynamic polyhedra resembles the identical rotational directionality of each facial unit in some virus capsids. By controlling the vertex, we stereoselectively synthesised thermodynamic homo-directional FRP and kinetic hetero-directional products. This strategy could be extended to other multifarious 2D asymmetric molecules to produce more complex Platonic and Archimedean polyhedra, which could be used for molecular recognition, chiral separation or asymmetric catalysis. We anticipate our assay to be a starting point towards more sophisticated molecular polyhedra with rotational faces, providing a strategy to mimic the sophistication of viruses.

## Methods

### Synthesis of the FRP

Vicinal diamine (6 eq) in anhydrous toluene and TFA (0.025 eq) were added to a schlenk flask (1 l) and stirred until dissolved. **TR** (4 eq) in toluene (400 to 600 ml) was added dropwise to the flask within 24 h. The solution was kept stirring for another 24 h. Solvents were evaporated under vacuum and solid was used for chiral separation and crystal growth without further purification. General experimental information and characterization details can be found in the Supplementary Methods.

### NMR and MS characterization

^1^H and ^13^C nuclear magnetic resonance (NMR) spectra were recorded on a Bruker AVIII-500 spectrometer (500 and 125 MHz, respectively) or Bruker AVIII-850 spectrometer at 298 K (850 and 213 MHz, respectively) and are reported relative to residual solvent signals. Matrix-assisted laser desorption/ionization time of flight mass spectra (MALDI-TOF MS) were collected on a Bruker microflex LT-MS with 2,4,6-trihydrotyacetophenane (0.05 M in methanol) as matrix. High-resolution mass spectra (HRMS) were collected on a Bruker En Apex Ultra 7.0T FT-MS.

### Single-crystal X-ray diffraction

Single crystal X-ray diffraction data were collected on Rigaku SuperNova X-Ray single crystal diffractometer using Cu Kα (λ=1.54184 Å) micro-focus X-ray sources at 100 K. The raw data were collected and reduced by CrysAlisPro software, the structures were solved by SHELXS or SHELXT and refined by SHELXL. Solution and refinement procedures are presented in the Supplementary Methods and specific details are compiled in Supplementary Table 1 and Supplementary Figs 18–22. Crystallographic information files are provided as Supplementary Data (Supplementary Data 1 for **1**, Supplementary Data 2 for (*AAAA*)-**2**, Supplementary Data 3 for (*CAAA*)-**2**, Supplementary Data 4 for (*CCCC*)-**3**, Supplementary Data 5 for (*CCCA*)-**3**).

### HPLC and CD characterization

HPLC analyses were performed on a Shimadzu LC-16A instrument, using Daicel Chiralcel IE Columns. FRP **1** was separated by HPLC in the mobile phase of hexane:ethyl acetate=83:17 (v/v) with 0.1% diethylamine, and FRP **2** was separated in the mobile phase of hexane:ethanol=75:25 (v/v) with 0.1% diethylamine. CD spectra were collected on JASCO J-810 circular dichroism spectrometer at 298 K. All CD spectra of the separated enantiomers were measured in hexane.

### Kinetics of the synthesis

**TR** (122 mg, 0.160 mmol), (R,R)-CHDA (14.4 mg, 0.240 mmol) and TFA (1.37 mg, 0.0120, mmol) were reacted in toluene (100 ml) at 298 K. At certain time intervals, the reaction solution were drawn out and measured by HPLC in the mobile phase of hexane:ethanol=75:25 (v/v) with 0.1% diethylamine. The experimental details are presented in the Supplementary Methods and HPLC spectra are compiled in Supplementary Figs 23–35.

### Computational methods

All the structural optimization and energy calculation were carried out in Materials Studios 7.0 by using the COMPASS II force field. The CD spectra were calculated at ZINDO semi-empirical level with Gaussian 09. Details of the method are provided in the Supplementary Methods. Coordinates of all optimized structures are provided in Supplementary Data 6.

### Data availability

Crystallographic data in this study were deposited at the Cambridge Crystallographic Data Centre with the accession codes (CCDC 1063965, 1406534, 1406540, 1406657, and 1407221). The authors declare that all other data supporting the findings of this study are available within the article and its Supplementary Information files or available from the authors upon reasonable request.

## Additional information

**How to cite this article:** Wang, X. *et al.* Assembled molecular face-rotating polyhedra to transfer chirality from two to three dimensions. *Nat. Commun.* 7:12469 doi: 10.1038/ncomms12469 (2016).

## Supplementary Material

Supplementary Information Supplementary Figures 1-35, Supplementary Table 1, Supplementary Methods and Supplementary References

Supplementary Data 1 Cif file of octahedron 1

Supplementary Data 2 Cif file of octahedron (AAAA)-2

Supplementary Data 3 Cif file of octahedron (CAAA)-2

Supplementary Data 4 Cif file of octahedron (CCCA)-3

Supplementary Data 5 Cif file of octahedron (CCCC)-3

Supplementary Data 6 Coordinates of all optimized structures

## Figures and Tables

**Table 1:** Energies of octahedra 1 and 2 calculated with COMPASS II force field.

Structure*	Valence energy† (diag. terms)	Valence energy‡ (cross terms)	Non-bond energy§=(van der Waals+electrostatic)			Total energy||
(*CCCC*)-**1**	726.711	−97.164	38.566	**3.093**	35.473	**668.112**
(*CCCA*)-**1**	731.605	−98.323	47.061	**12.828**	34.233	**680.343**
(*CCAA*)-**1**	732.137	−99.082	58.627	**24.938**	33.690	**691.682**
(*CAAA*)-**1**	731.607	−98.324	47.060	**12.826**	34.234	**680.343**
(*AAAA*)-**1**	726.717	−97.163	38.558	**3.087**	35.471	**668.112**
						
(*CCCC*)-**2**	645.467	−122.125	81.931	**49.480**	32.451	**605.273**
(*CCCA*)-**2**	650.196	−124.709	74.355	**39.606**	34.749	**599.842**
(*CCAA*)-**2**	650.221	−121.005	63.485	**28.970**	34.516	**592.702**
(*CAAA*)-**2**	649.340	−121.103	57.521	**19.378**	38.143	**585.757**
(*AAAA*)-**2**	641.876	−118.330	44.825	**8.590**	36.235	**568.371**
						
(*CCCC*)-**3**	641.875	−118.331	44.826	**8.591**	36.235	**568.371**
(*CCCA*)-**3**	647.413	−119.395	53.218	**17.367**	35.851	**581.236**
(*CCAA*)-**3**	650.219	−121.006	63.489	**28.972**	34.517	**592.702**
(*CAAA*)-**3**	650.197	−124.708	74.353	**39.605**	34.748	**599.842**
(*AAAA*)-**3**	645.478	−122.127	81.923	**49.473**	32.449	**605.273**

^*^Structures were constructed on the basis of the crystal structures and adjusted to follow the symmetries of *T* (*CCCC* and *AAAA*), *C*_*3*_ (*CCCA* and *CAAA*) and *C*_*2*_ (*CCAA*). The constructed structures were calculated with the COMPASS II force field.

^†^Valence energy (diag. terms) contains the contributions of bond, angle, torsion, and inversion.

^‡^Valence energy (cross terms) contains the contributions of stretch-stretch, stretch-bend-stretch, stretch-torsion-stretch, separated-stretch-stretch, torsion-stretch, bend-bend, torsion-bend-bend and bend-torsion-bend.

^§^Non-bond energy contains the contributions of van der Waals and electrostatic interactions.

^||^All of the energies are reported in kcal mol^−1^. The differences among total energies of each isomers are mostly rooted in the differences in van der Waals interaction, as highlighted in two bold entries.
