# Supplementary material for: Assembled molecular face-rotating polyhedra to transfer chirality from two to three dimensions
Source: Nat Commun. 2016 Aug 24;7:12469. doi: 10.1038/ncomms12469 (PMC4999497; doi:10.1038/ncomms12469)
Supplement: Supplementary Information — Supplementary Figures 1-35, Supplementary Table 1, Supplementary Methods and Supplementary References [file ncomms12469-s1.pdf]

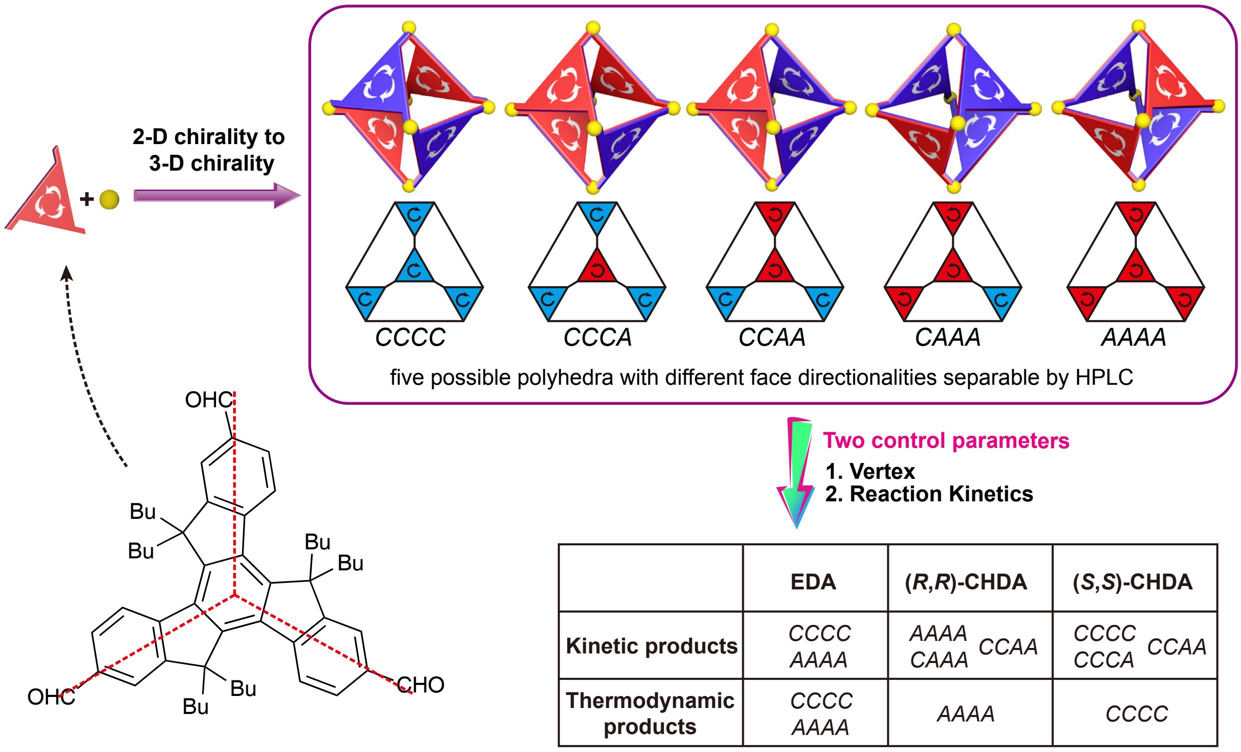

	EDA	(*R*,*R*)-CHDA	(*S*,*S*)-CHDA
**Kinetic products**	*CCCC* *AAAA*	*AAAA* *CAAA* *CCAA*	*CCCC* *CCCA* *CCAA*
**Thermodynamic products**	*CCCC* *AAAA*	*AAAA*	*CCCC*

Supplementary Figure 1. Five possible octahedra with different facial directionalities controlled by three parameters. The molecular structure of the truxene building block is shown in the bottom left. The cartoon and topological representations of the five possible octahedra are shown in the top right. The product distributions of octahedra **1** and **2** are shown in the bottom right.

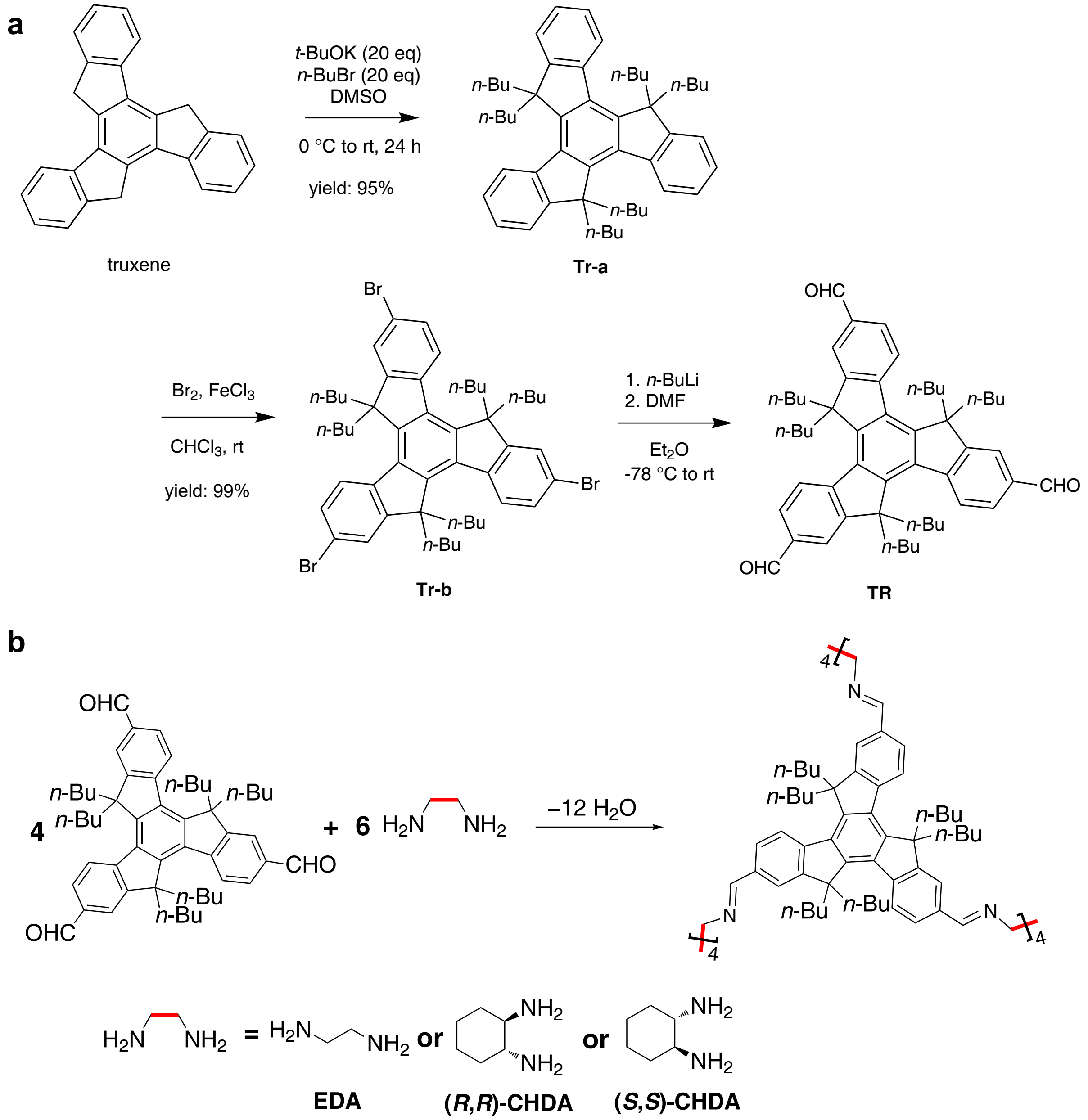

Supplementary Figure 2. Representative procedures for the syntheses of building block TR (**a**) and octahedra (**b**).

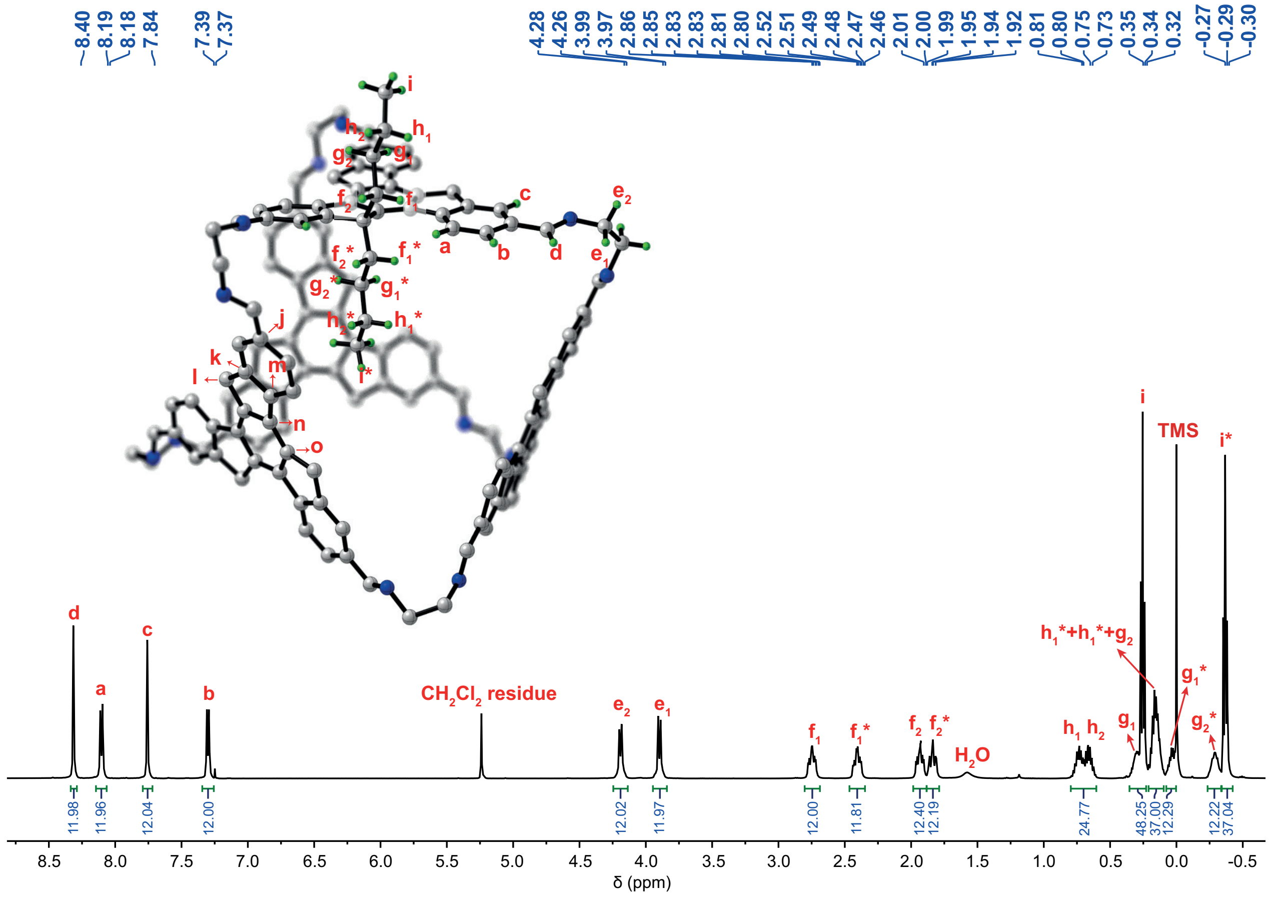

Supplementary Figure 3. ^{1}H NMR spectrum of **1** (only one butyl chain is shown for clarity).

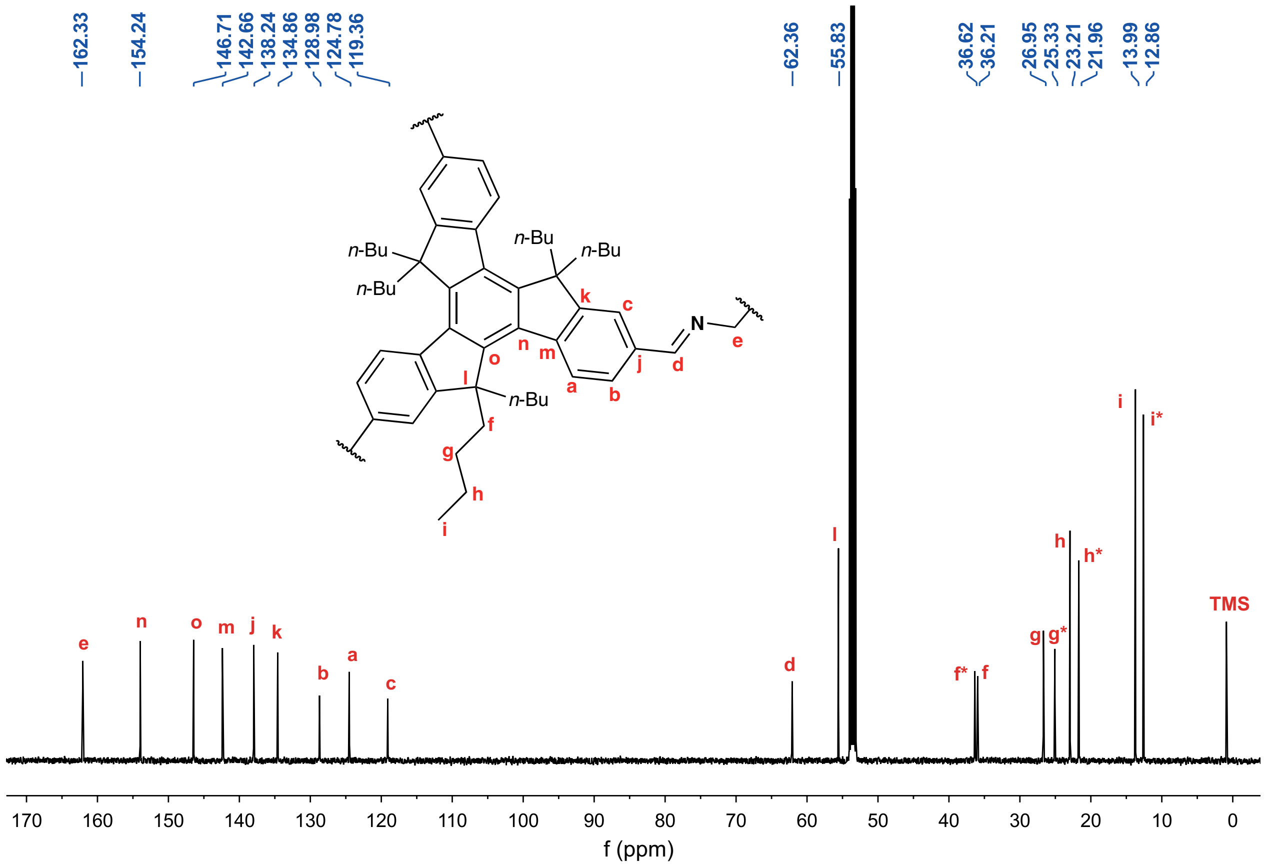

Supplementary Figure 4. ^{13}C NMR spectrum of **1**.

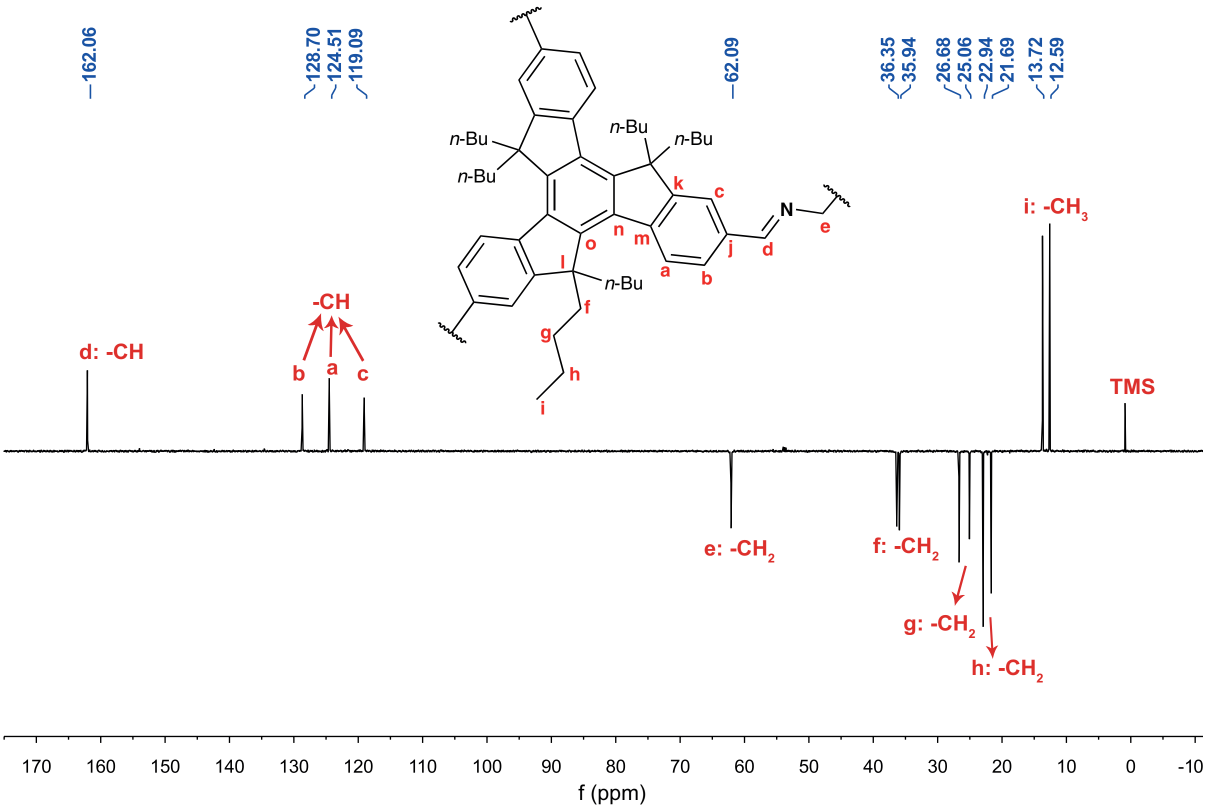

Supplementary Figure 5. Distortionless Enhancement by Polarization Transfer (DEPT)-135 spectrum of **1**.

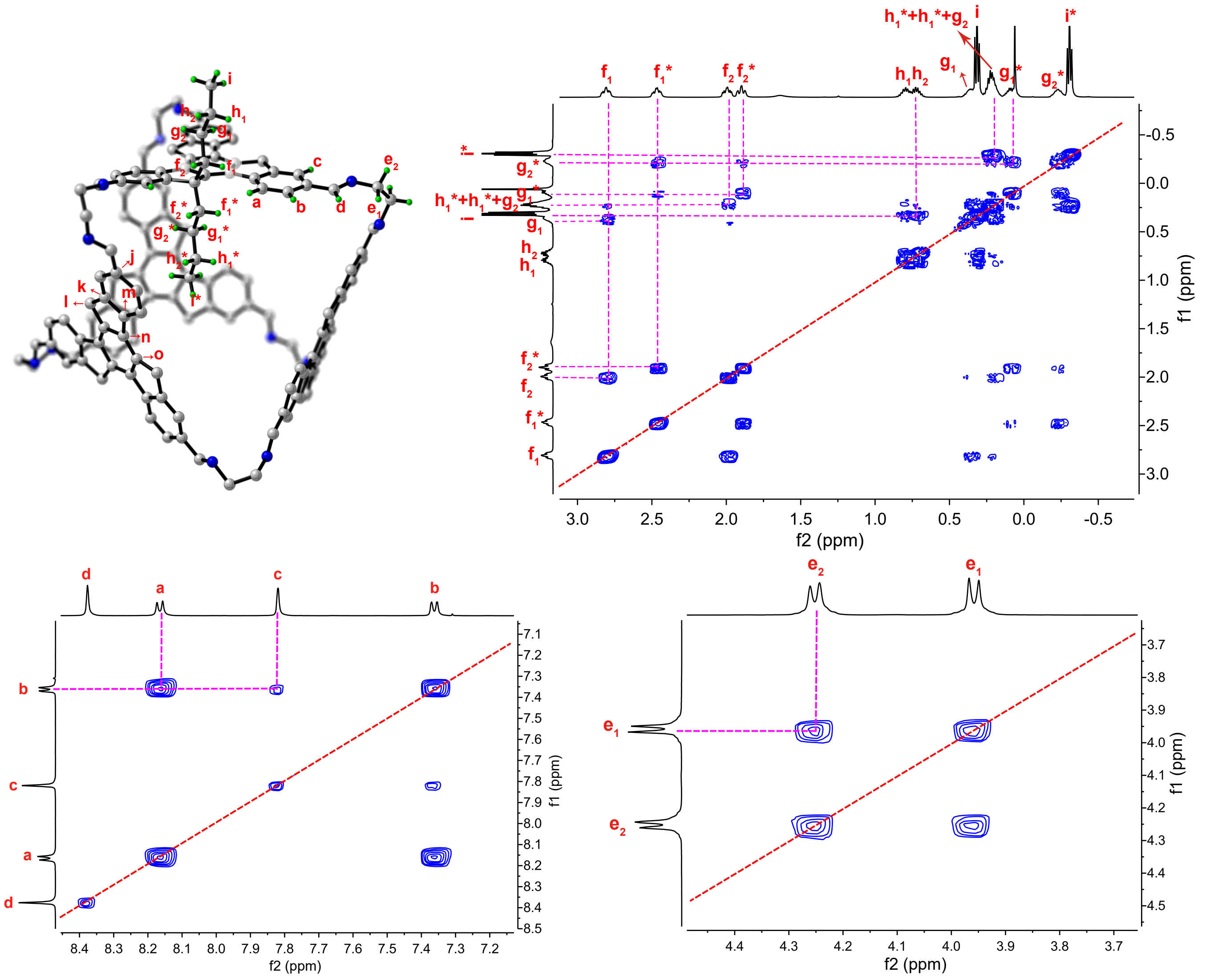

Supplementary Figure 6. HH correlation spectroscopy (COSY) spectrum of **1**.

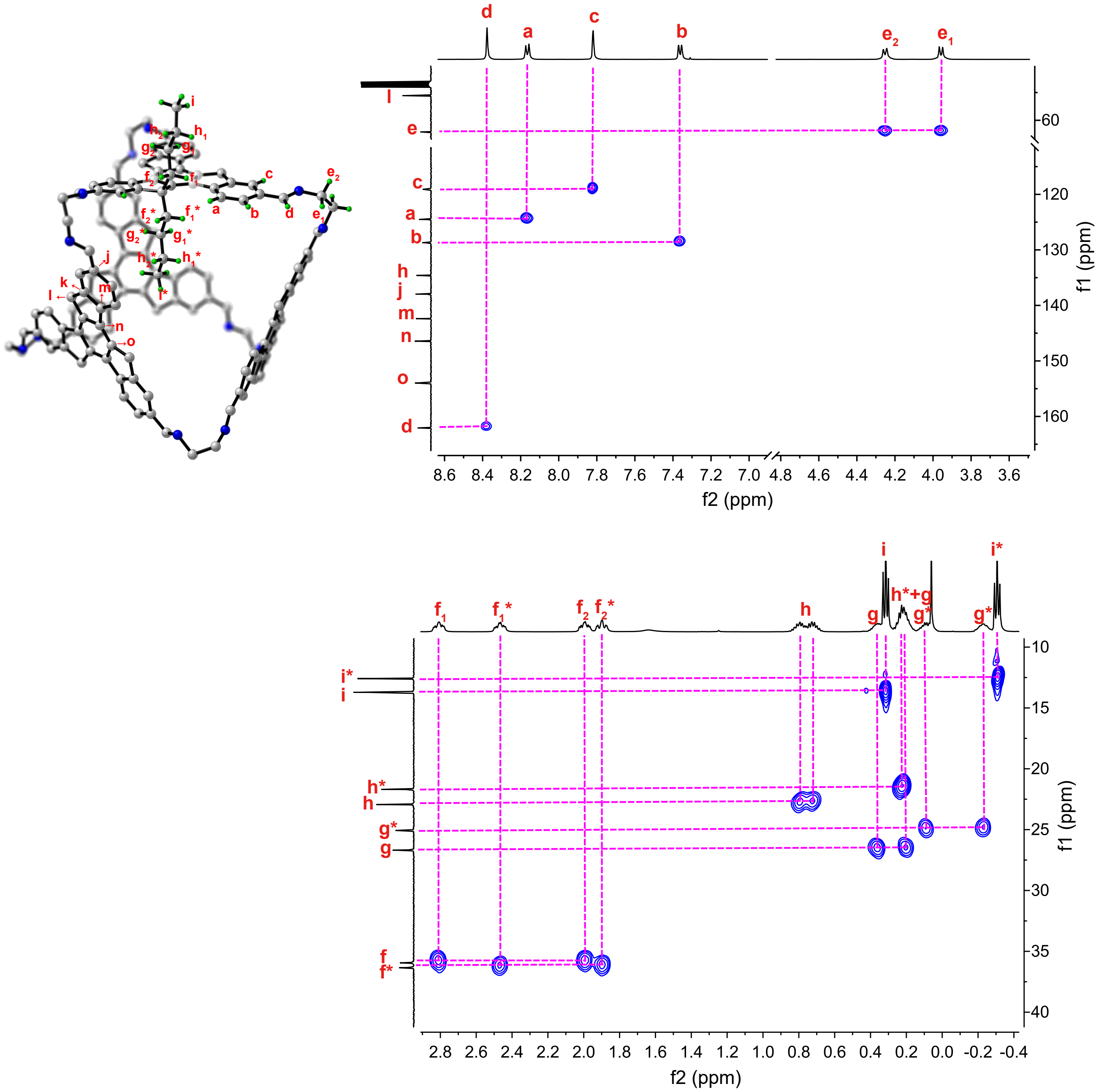

Supplementary Figure 7. Heteronuclear single-quantum correlation (HSQC) spectrum of **1**.

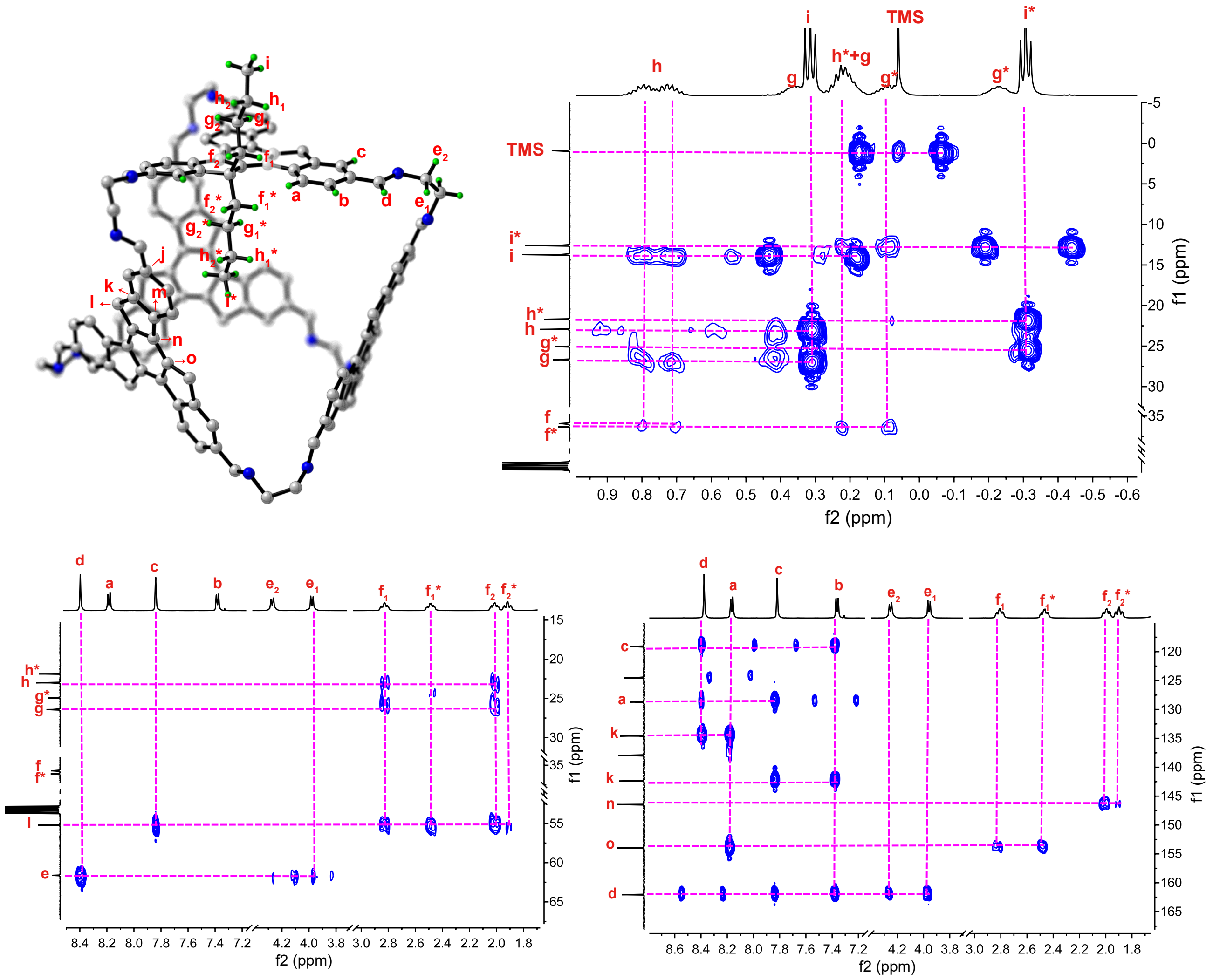

Supplementary Figure 8. ^{1}H detected heteronuclear multiple bond correlation (HMBC) spectrum of **1**.

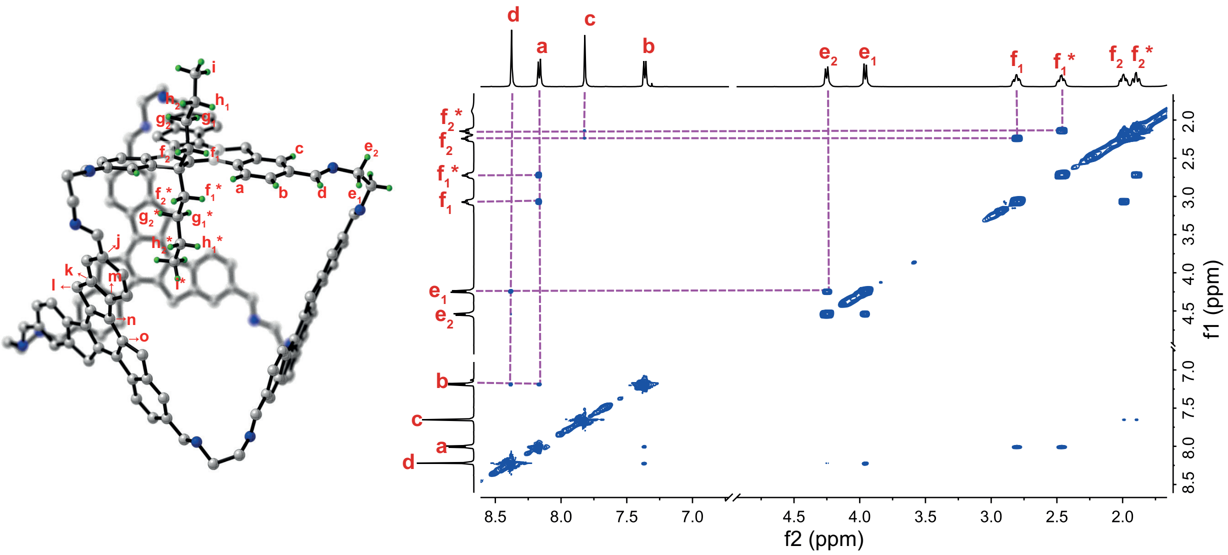

Supplementary Figure 9. Nuclear Overhauser enhancement spectroscopy (NOESY) spectrum of **1**.

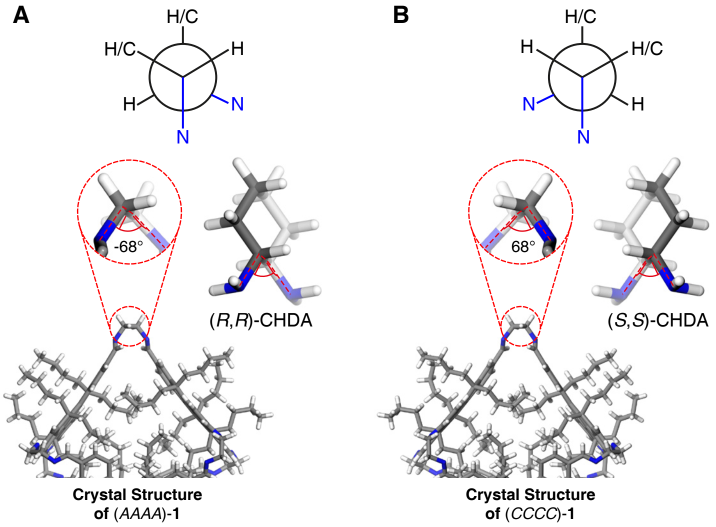

Supplementary Figure 10. Conformation of the EDA vertex in octahedron **1**. **a**, The crystal structure of (*AAAA*)-**1** shows the EDA vertices are in gauche conformation with a torsion angle of -68° between the two amino groups, resembling the configuration of (*R*,*R*)-CHDA. **b**, The crystal structure of (*CCCC*)-**1** shows a mirror-like vertex conformation with a torsion angle of -68° between the two amino groups, resembling the configuration of (*S*,*S*)-CHDA.

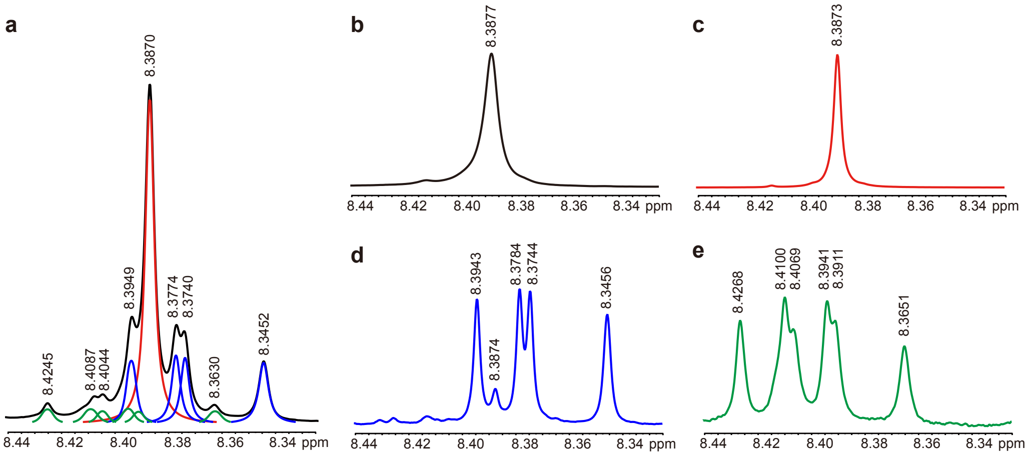

Supplementary Figure 11. ^{1}H NMR spectra of octahedron **2**. **a**, NMR spectra of crude **2** (black line) synthesised at 25 ºC for 24 hours. The red, blue, and green lines are deconvoluted Lorentzian peaks of (*AAAA*)-**2**, (*CAAA*)-**2**, and (*CCAA*)-**2**, respectively. **b**, NMR spectrum of **2** heated at 110 ºC for 48 hours. **c**–**e**, NMR spectra of (*AAAA*)-**2** (**c**), (*CAAA*)-**2** (**d**), and (*CCAA*)-**2** (**e**), which were separated by HPLC, as shown in Fig. 2f.

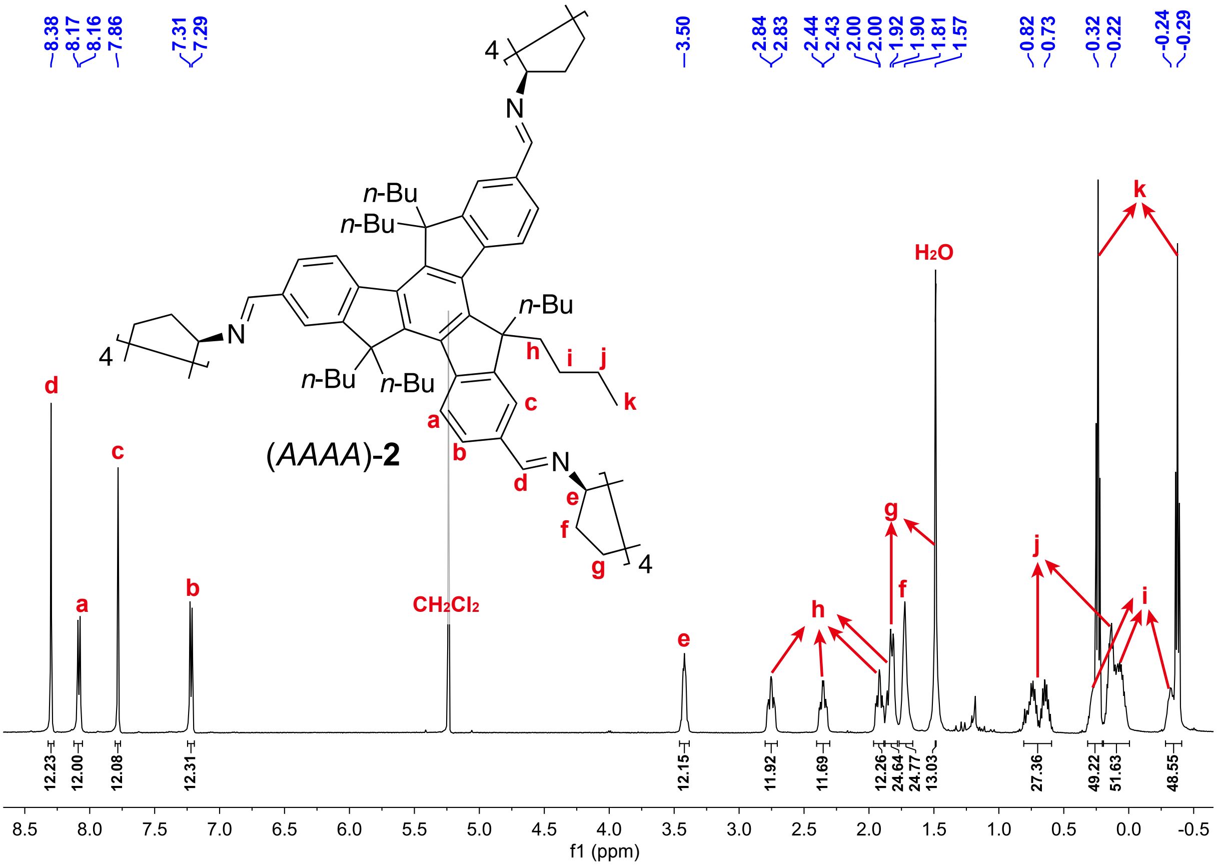

Supplementary Figure 12. ^{1}H NMR spectrum of **1**

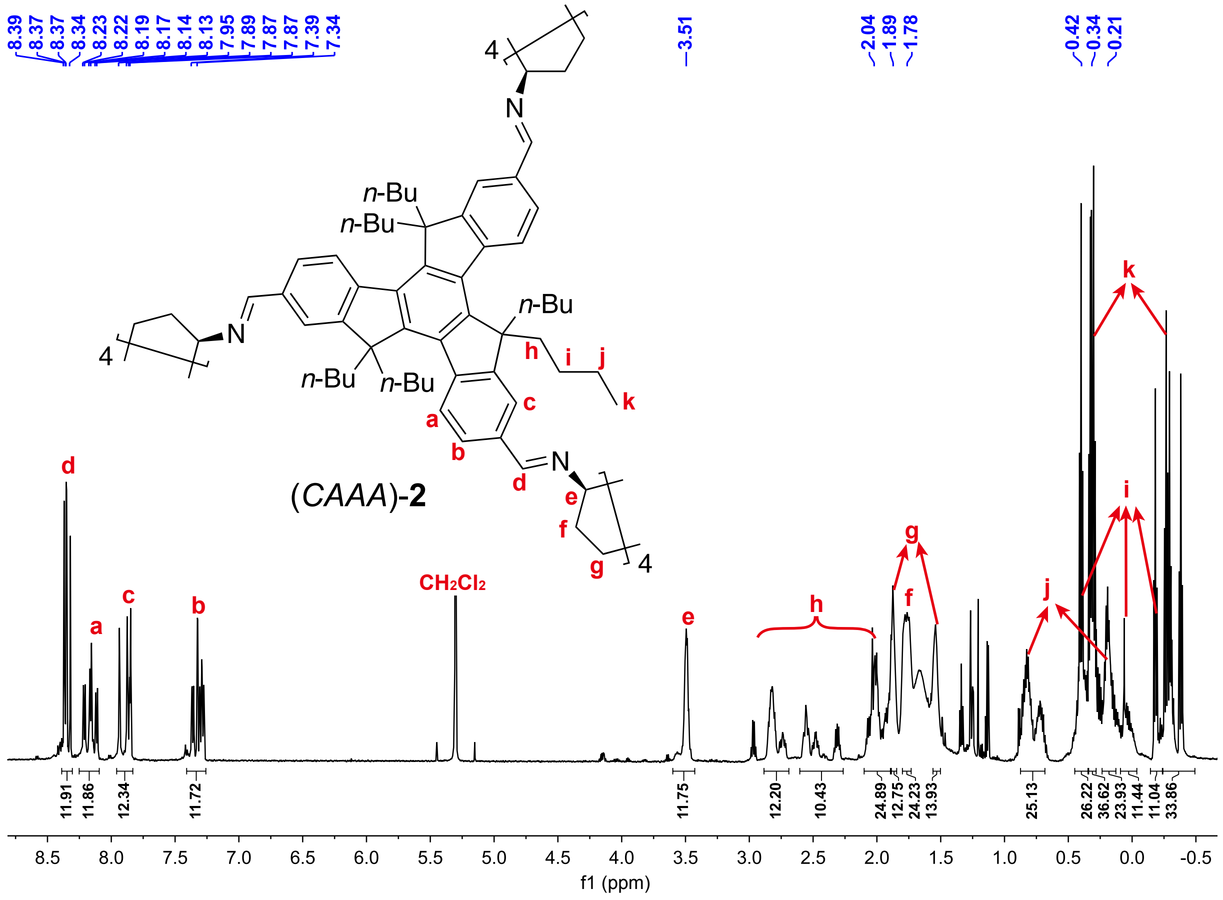

Supplementary Figure 13. ^{1}H NMR spectrum of (*CAAA*)-**2**

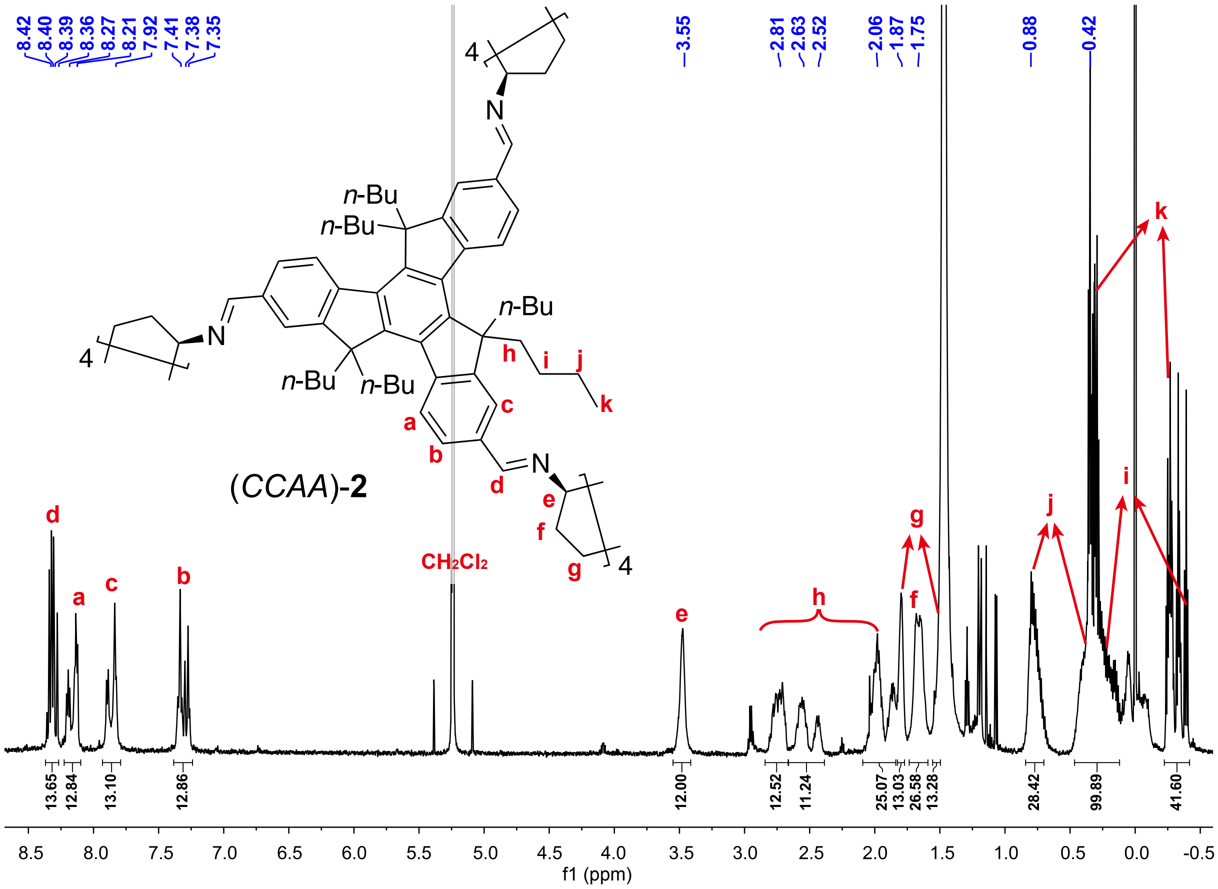

Supplementary Figure 14. ^{1}H NMR spectrum of (*CCAA*)-**2**

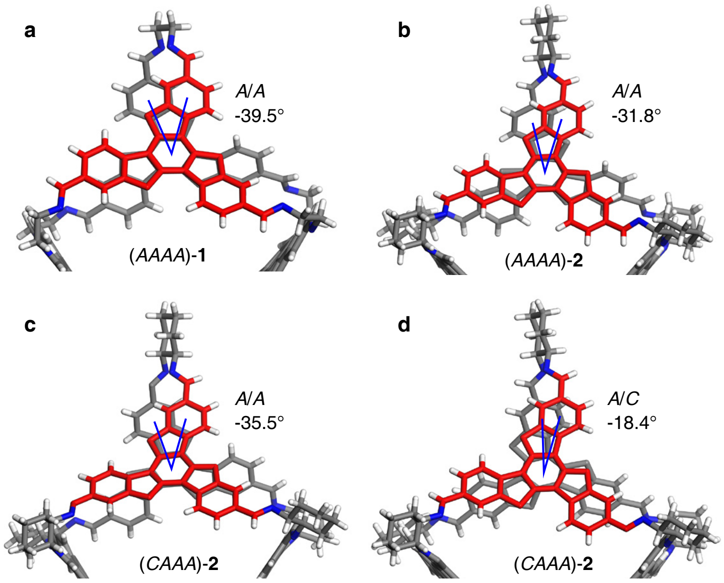

Supplementary Figure 15. Alternate angles of the truxene cores in different octahedra. **a**, The alternate angle between two clockwise faces in (*AAAA*)-**1** was -39.5° (left-handed configuration) according to single-crystal X-ray diffraction analysis. **b**, The alternate angle between two clockwise faces in (*AAAA*)-**2** was -31.8°. **c**, The alternate angle between two clockwise faces in (*CAAA*)-**2** was -35.5°. **d**, The alternate angle between a clockwise face and an anticlockwise face in (*CAAA*)-**2** was -18.4°.

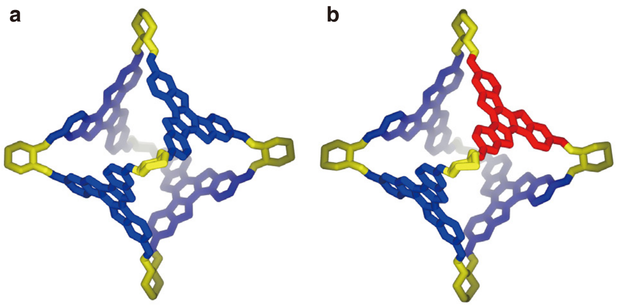

Supplementary Figure 16. Single-crystal structure of octahedron **3**. Single-crystal structures of the thermodynamic (*CCCC*)-**3** (**a**) and kinetically stable (*CCCA*)-**3** (**b**). The clockwise and anticlockwise faces are indicated in red and blue, respectively.

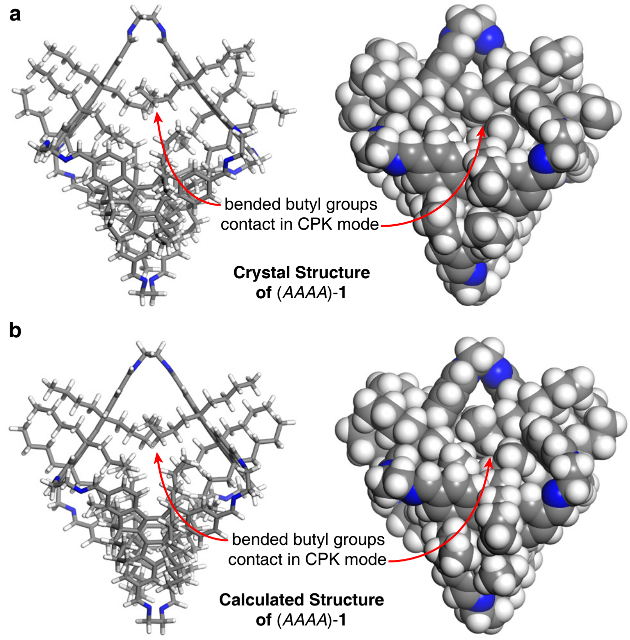

Supplementary Figure 17. Conformations of the butyl groups in (*AAAA*)-**1**. **a**, The crystal structure of (*AAAA*)-**1** shows that the butyl groups inside the octahedron are bended because of non-covalent repulsive interactions and that they contact each other in the CPK mode, whereas the exterior butyl groups remain straight. **b**, The calculated structure of (*AAAA*)-**1** indicates that the conformations of the butyl groups are similar to those in the crystal structure.

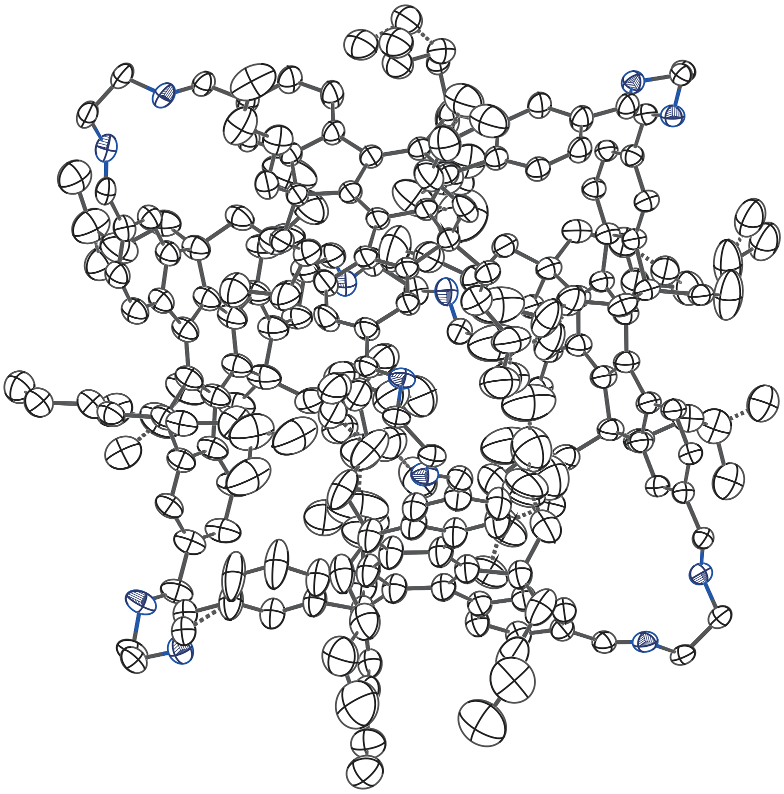

Supplementary Figure 18. ORTEP drawing of **1** (displacement ellipsoids for all non-H atoms at the 50% probability level).

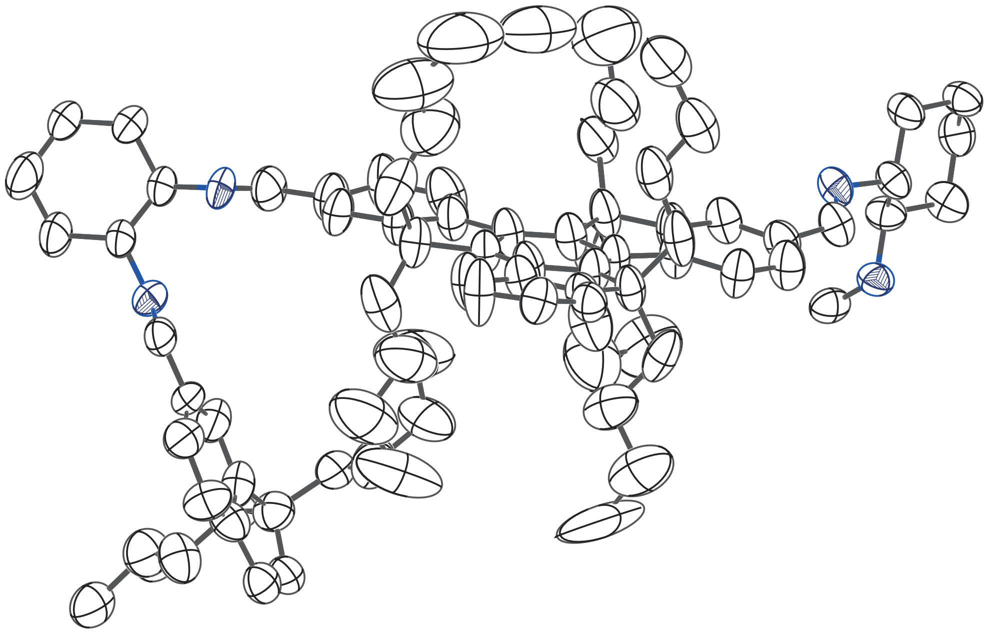

Supplementary Figure 19. ORTEP drawing of (*AAAA*)-**2** (displacement ellipsoids for all non-H atoms at the 50% probability level).

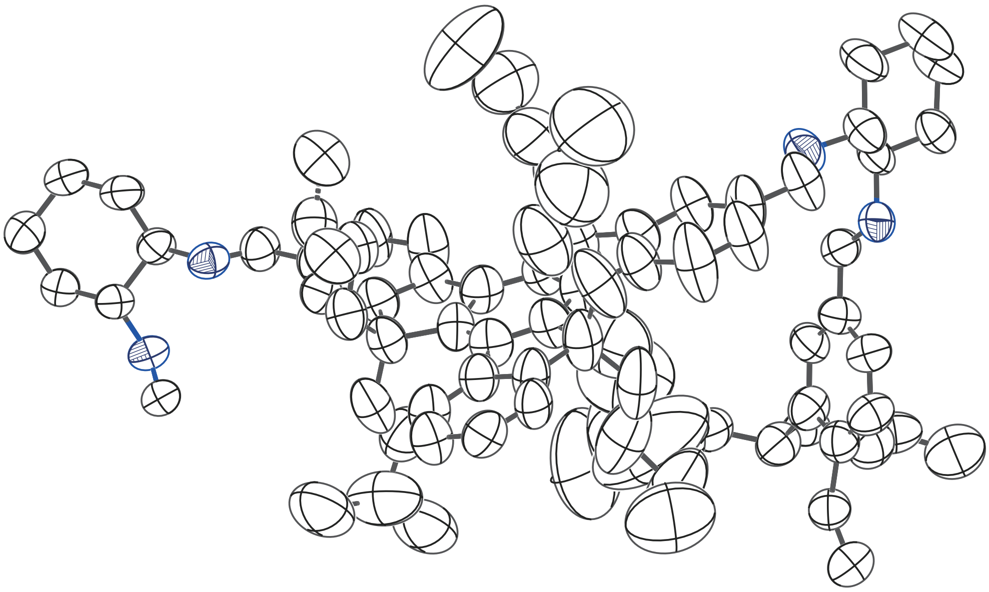

Supplementary Figure 20. ORTEP drawing of (*CAAA*)-**2** (displacement ellipsoids for all non-H atoms at the 50% probability level).

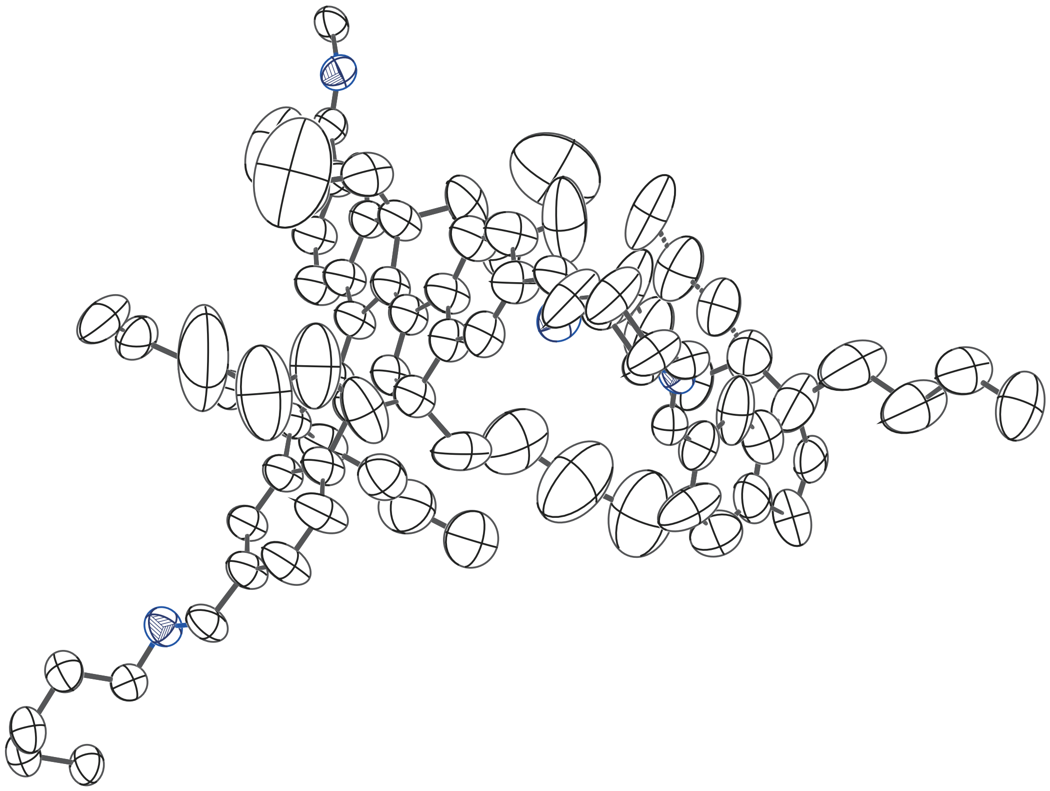

Supplementary Figure 21. ORTEP drawing of (*CCCC*)-**3** (displacement ellipsoids for all non-H atoms at the 50% probability level).

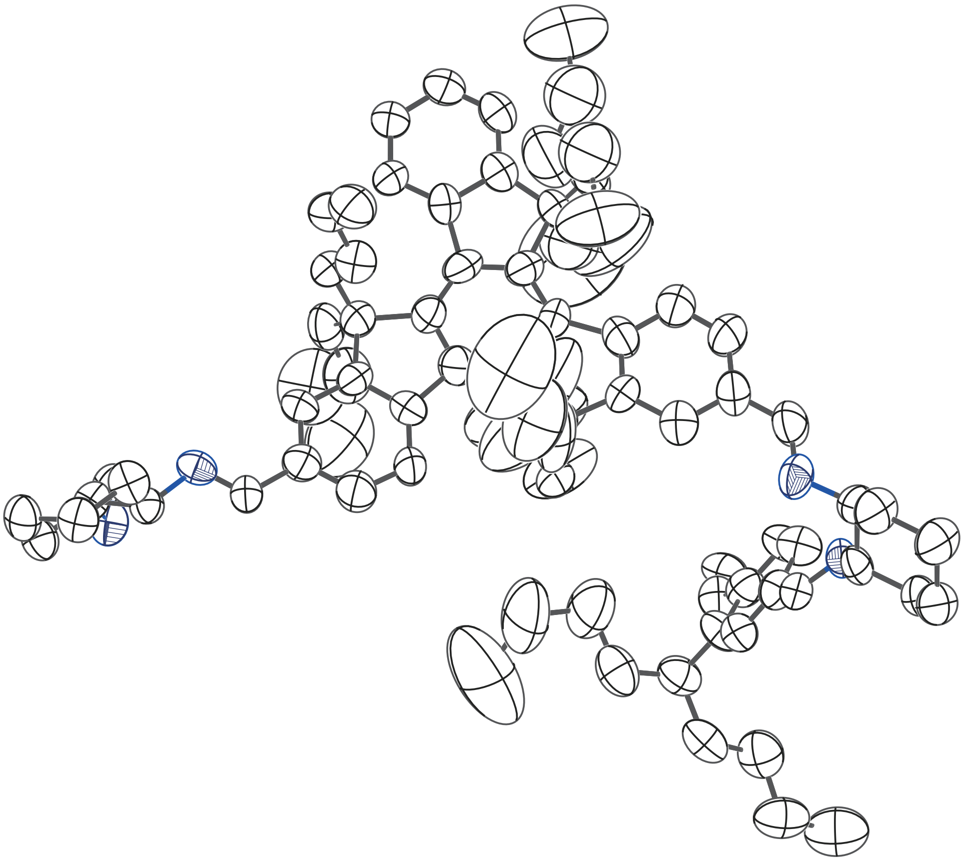

Supplementary Figure 22. ORTEP drawing of (*CCCA*)-**3** (displacement ellipsoids for all non-H atoms at the 50% probability level).

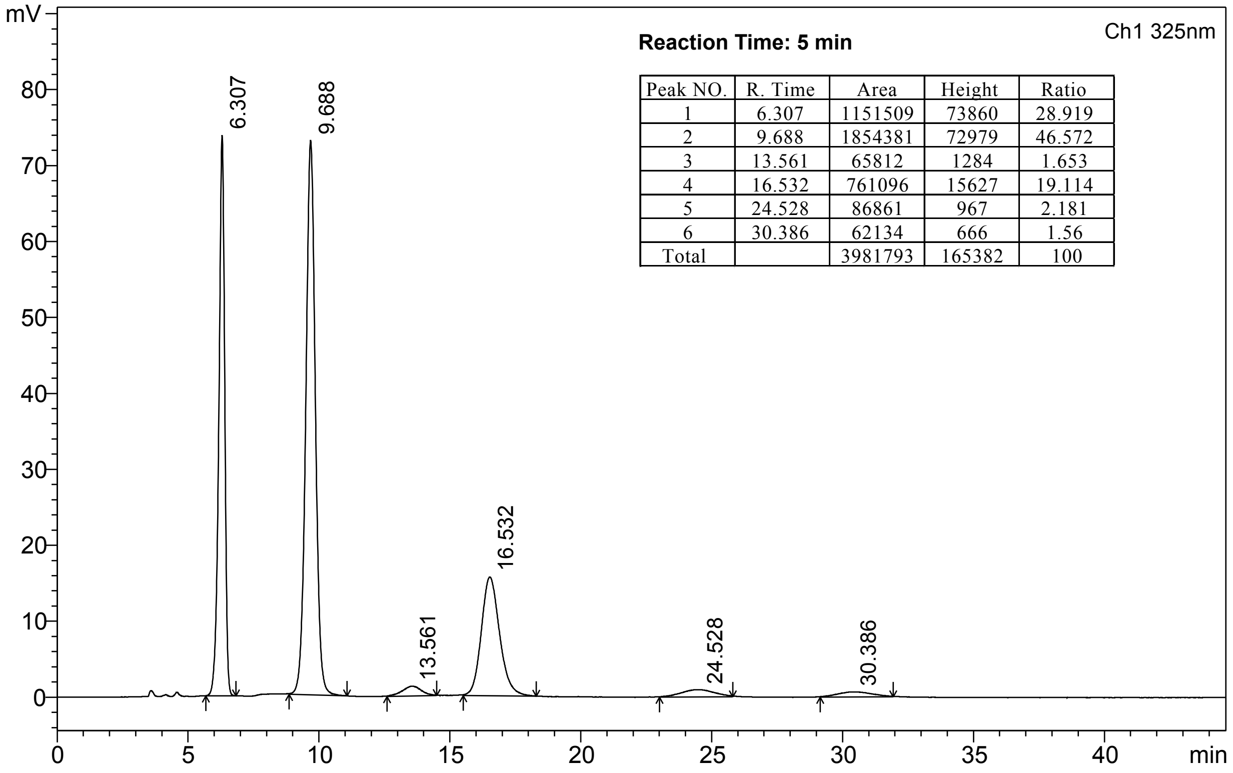

Peak NO.	R. Time	Area	Height	Ratio
1	6.307	1151509	73860	28.919
2	9.688	1854381	72979	46.572
3	13.561	65812	1284	1.653
4	16.532	761096	15627	19.114
5	24.528	86861	967	2.181
6	30.386	62134	666	1.56
Total		3981793	165382	100

Supplementary Figure 23. Time dependent HPLC spectra of the synthesis of **2** at 5 min.

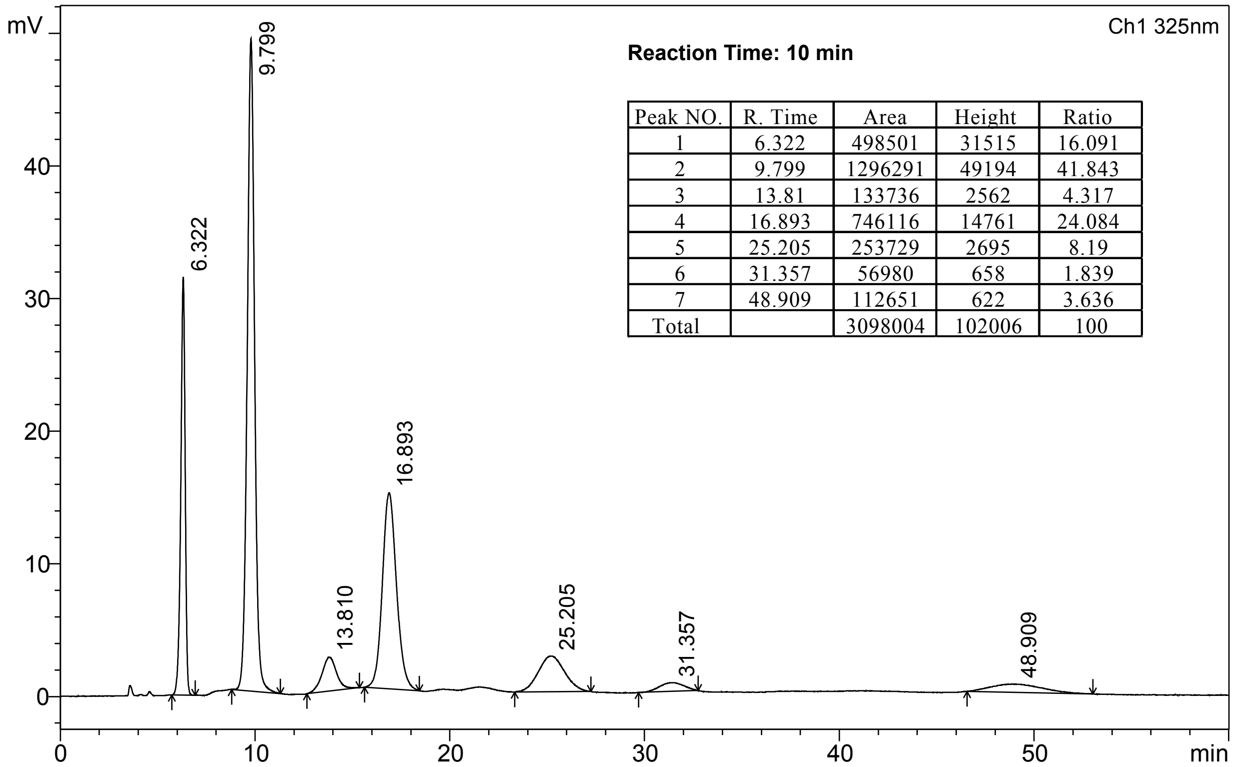

Peak NO.	R. Time	Area	Height	Ratio
1	6.322	498501	31515	16.091
2	9.799	1296291	49194	41.843
3	13.81	133736	2562	4.317
4	16.893	746116	14761	24.084
5	25.205	253729	2695	8.19
6	31.357	56980	658	1.839
7	48.909	112651	622	3.636
Total		3098004	102006	100

Supplementary Figure 24. Time dependent HPLC spectra of the synthesis of **2** at 10 min.

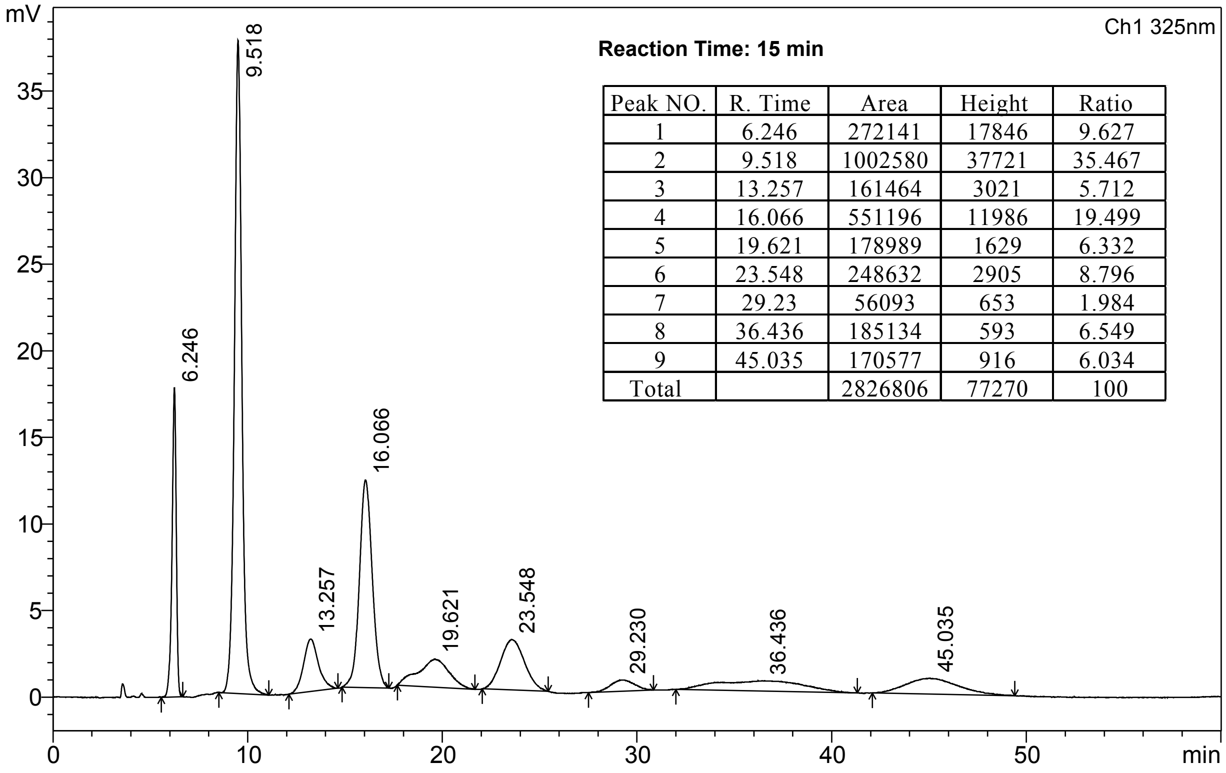

Peak NO.	R. Time	Area	Height	Ratio
1	6.246	272141	17846	9.627
2	9.518	1002580	37721	35.467
3	13.257	161464	3021	5.712
4	16.066	551196	11986	19.499
5	19.621	178989	1629	6.332
6	23.548	248632	2905	8.796
7	29.23	56093	653	1.984
8	36.436	185134	593	6.549
9	45.035	170577	916	6.034
Total		2826806	77270	100

Supplementary Figure 25. Time dependent HPLC spectra of the synthesis of **2** at 15 min.

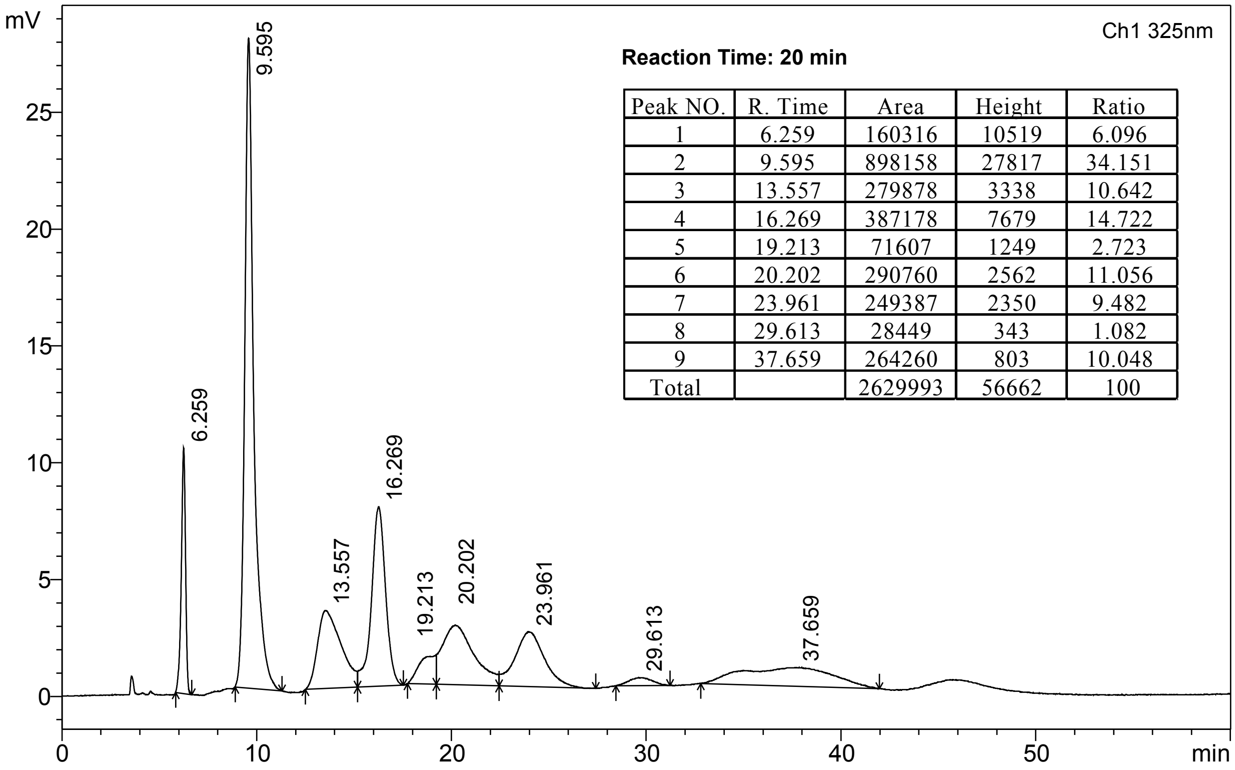

Peak NO.	R. Time	Area	Height	Ratio
1	6.259	160316	10519	6.096
2	9.595	898158	27817	34.151
3	13.557	279878	3338	10.642
4	16.269	387178	7679	14.722
5	19.213	71607	1249	2.723
6	20.202	290760	2562	11.056
7	23.961	249387	2350	9.482
8	29.613	28449	343	1.082
9	37.659	264260	803	10.048
Total		2629993	56662	100

Supplementary Figure 26. Time dependent HPLC spectra of the synthesis of **2** at 20 min.

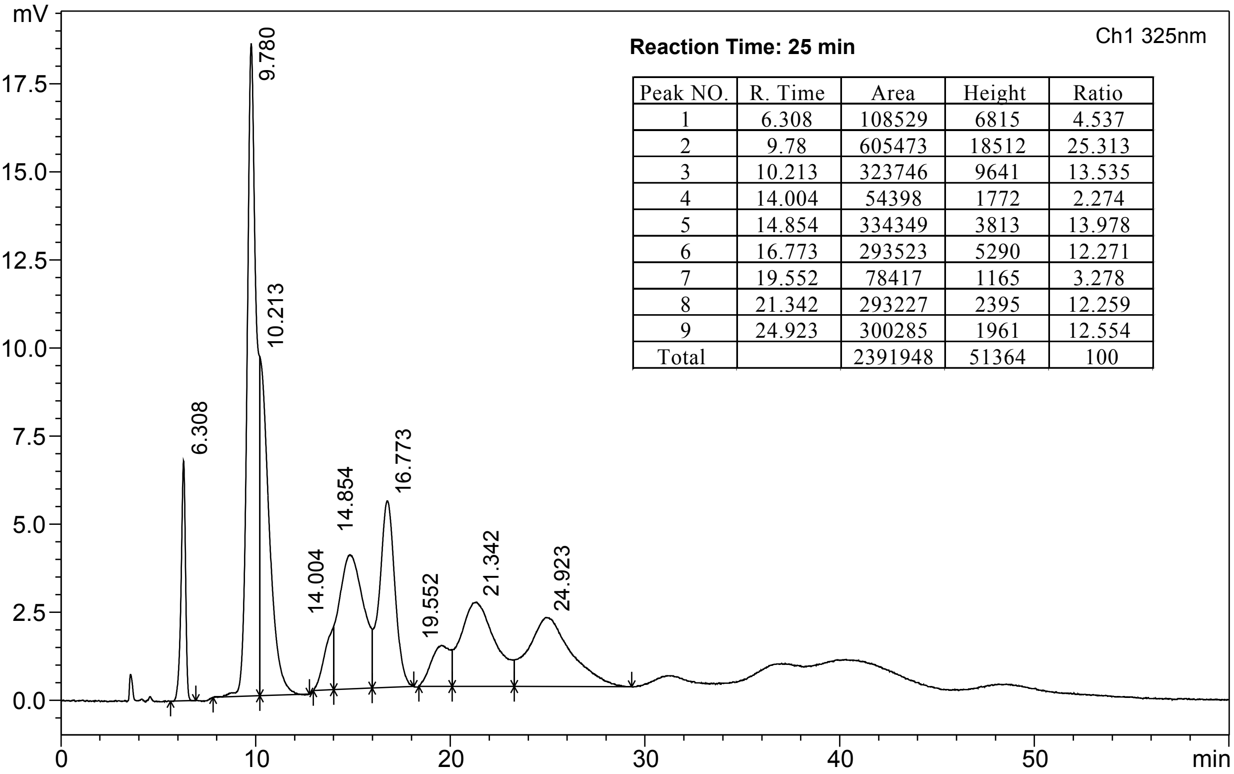

Peak NO.	R. Time	Area	Height	Ratio
1	6.308	108529	6815	4.537
2	9.78	605473	18512	25.313
3	10.213	323746	9641	13.535
4	14.004	54398	1772	2.274
5	14.854	334349	3813	13.978
6	16.773	293523	5290	12.271
7	19.552	78417	1165	3.278
8	21.342	293227	2395	12.259
9	24.923	300285	1961	12.554
Total		2391948	51364	100

Supplementary Figure 27. Time dependent HPLC spectra of the synthesis of **2** at 25 min.

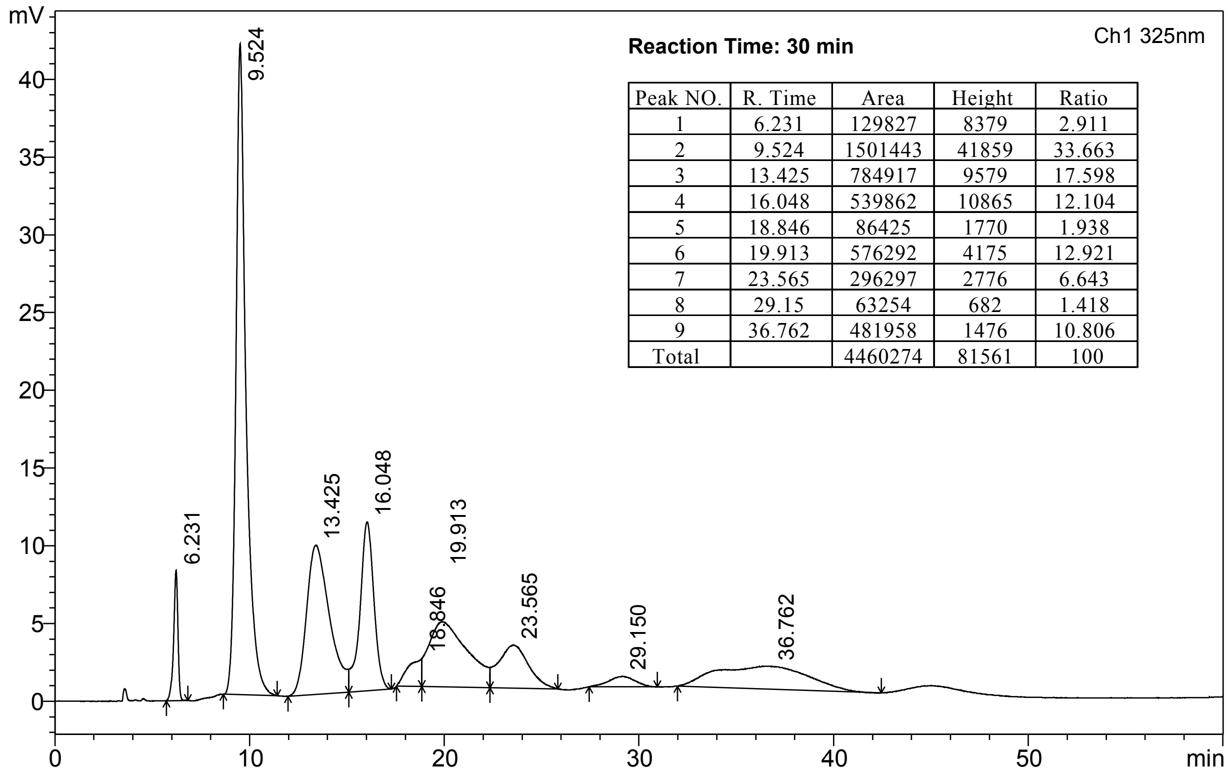

Peak NO.	R. Time	Area	Height	Ratio
1	6.231	129827	8379	2.911
2	9.524	1501443	41859	33.663
3	13.425	784917	9579	17.598
4	16.048	539862	10865	12.104
5	18.846	86425	1770	1.938
6	19.913	576292	4175	12.921
7	23.565	296297	2776	6.643
8	29.15	63254	682	1.418
9	36.762	481958	1476	10.806
Total		4460274	81561	100

Supplementary Figure 28. Time dependent HPLC spectra of the synthesis of **2** at 30 min.

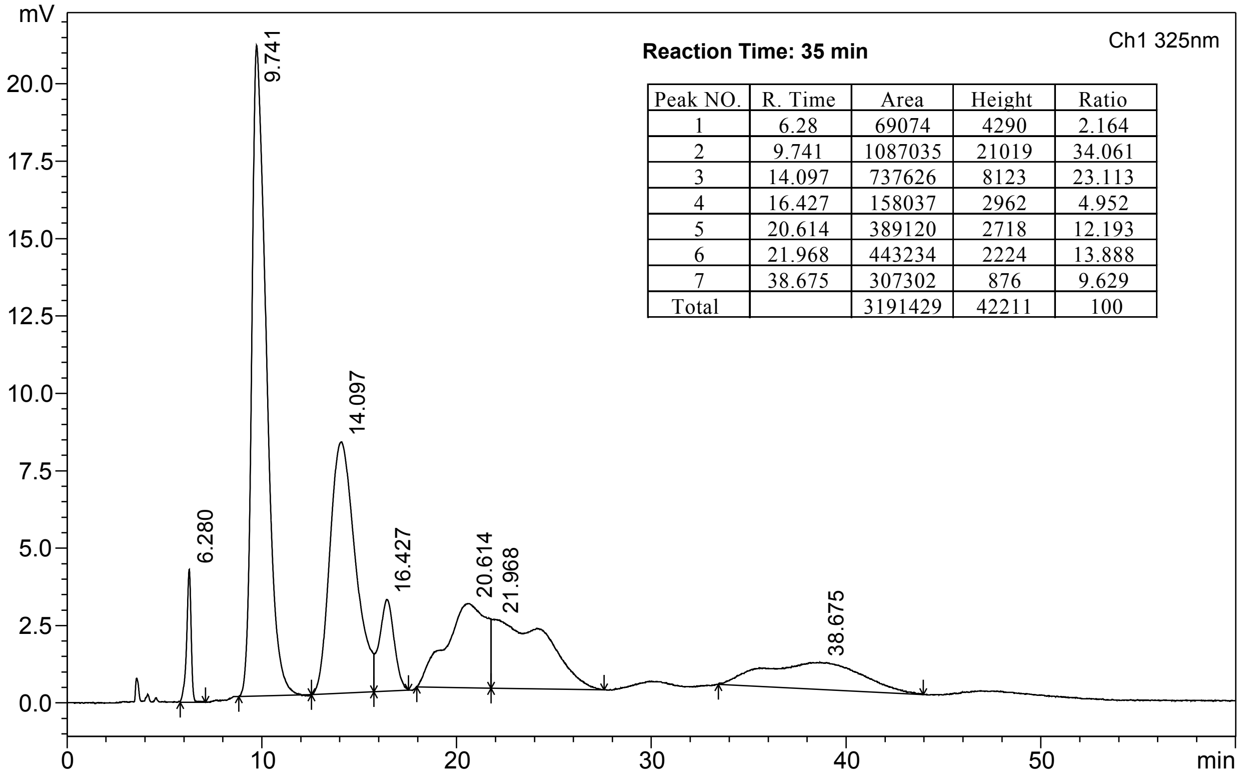

Peak NO.	R. Time	Area	Height	Ratio
1	6.28	69074	4290	2.164
2	9.741	1087035	21019	34.061
3	14.097	737626	8123	23.113
4	16.427	158037	2962	4.952
5	20.614	389120	2718	12.193
6	21.968	443234	2224	13.888
7	38.675	307302	876	9.629
Total		3191429	42211	100

Supplementary Figure 29. Time dependent HPLC spectra of the synthesis of **2** at 35 min.

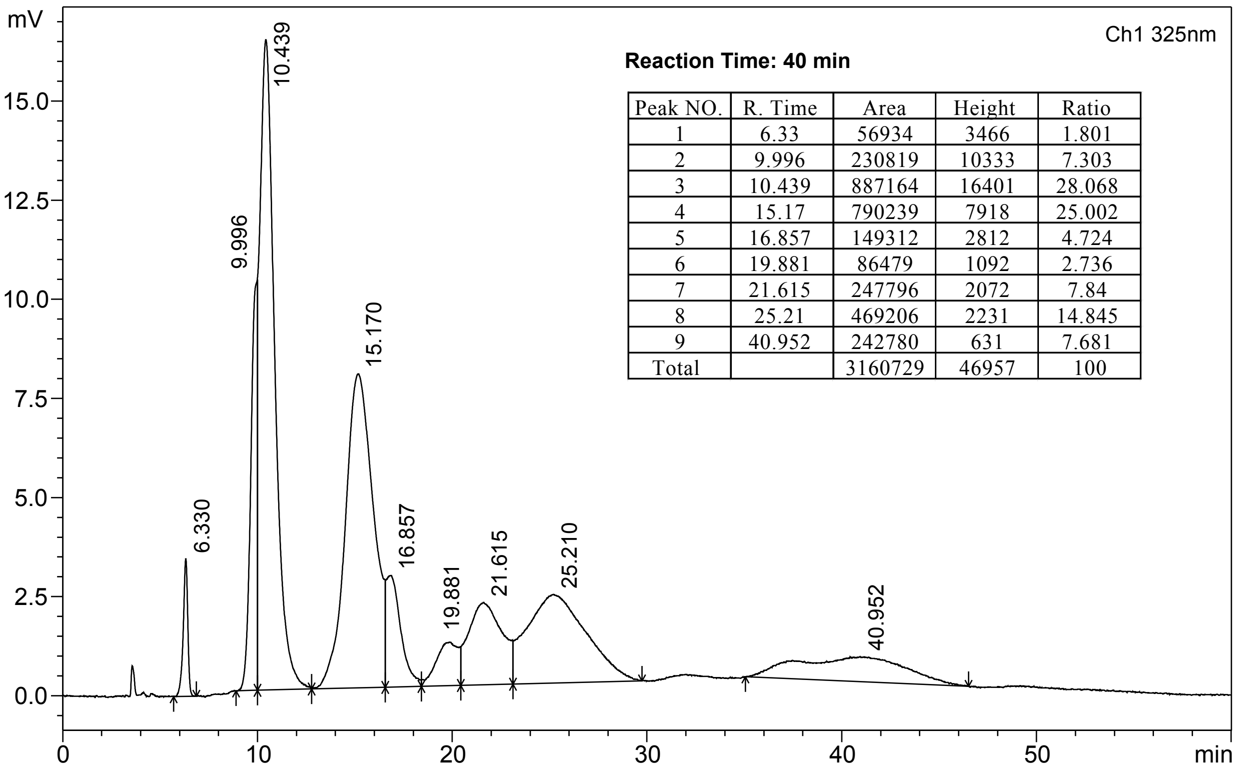

Peak NO.	R. Time	Area	Height	Ratio
1	6.33	56934	3466	1.801
2	9.996	230819	10333	7.303
3	10.439	887164	16401	28.068
4	15.17	790239	7918	25.002
5	16.857	149312	2812	4.724
6	19.881	86479	1092	2.736
7	21.615	247796	2072	7.84
8	25.21	469206	2231	14.845
9	40.952	242780	631	7.681
Total		3160729	46957	100

Supplementary Figure 30. Time dependent HPLC spectra of the synthesis of **2** at 40 min.

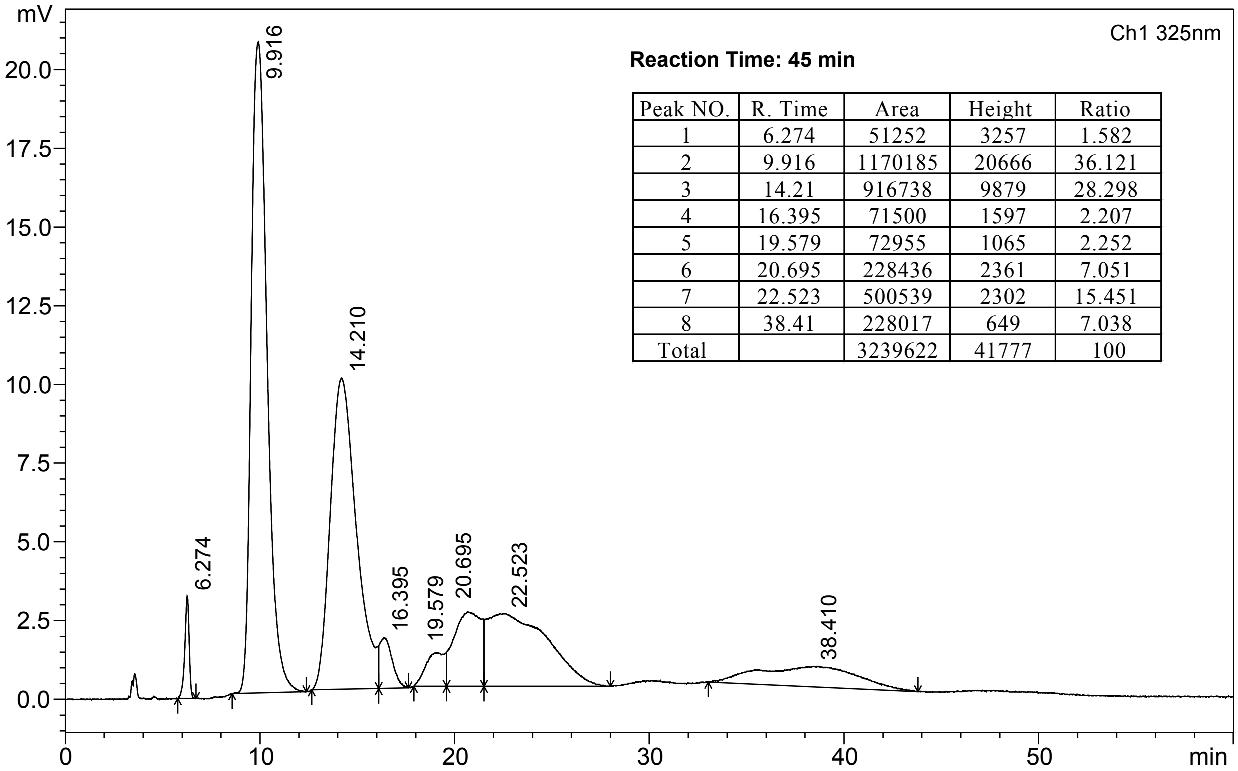

Peak NO.	R. Time	Area	Height	Ratio
1	6.274	51252	3257	1.582
2	9.916	1170185	20666	36.121
3	14.21	916738	9879	28.298
4	16.395	71500	1597	2.207
5	19.579	72955	1065	2.252
6	20.695	228436	2361	7.051
7	22.523	500539	2302	15.451
8	38.41	228017	649	7.038
Total		3239622	41777	100

Supplementary Figure 31. Time dependent HPLC spectra of the synthesis of **2** at 45 min.

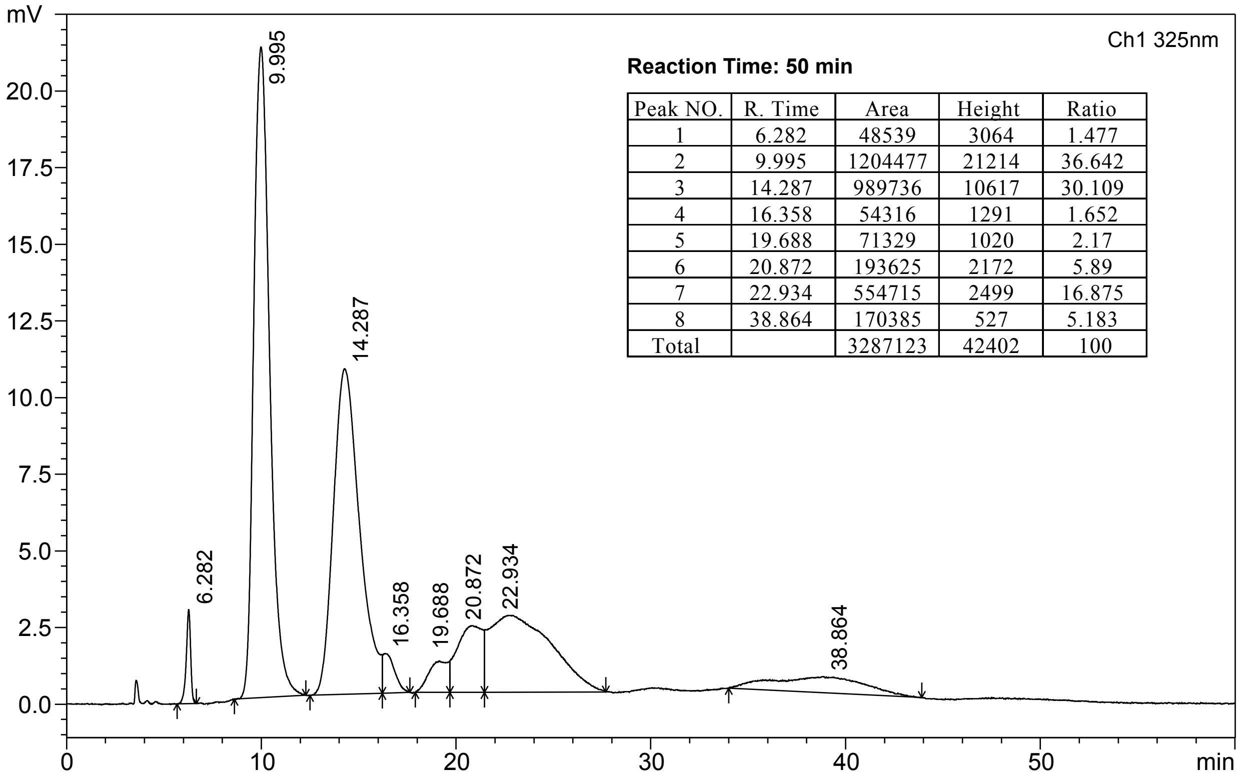

Peak NO.	R. Time	Area	Height	Ratio
1	6.282	48539	3064	1.477
2	9.995	1204477	21214	36.642
3	14.287	989736	10617	30.109
4	16.358	54316	1291	1.652
5	19.688	71329	1020	2.17
6	20.872	193625	2172	5.89
7	22.934	554715	2499	16.875
8	38.864	170385	527	5.183
Total		3287123	42402	100

Supplementary Figure 32. Time dependent HPLC spectra of the synthesis of **2** at 50 min.

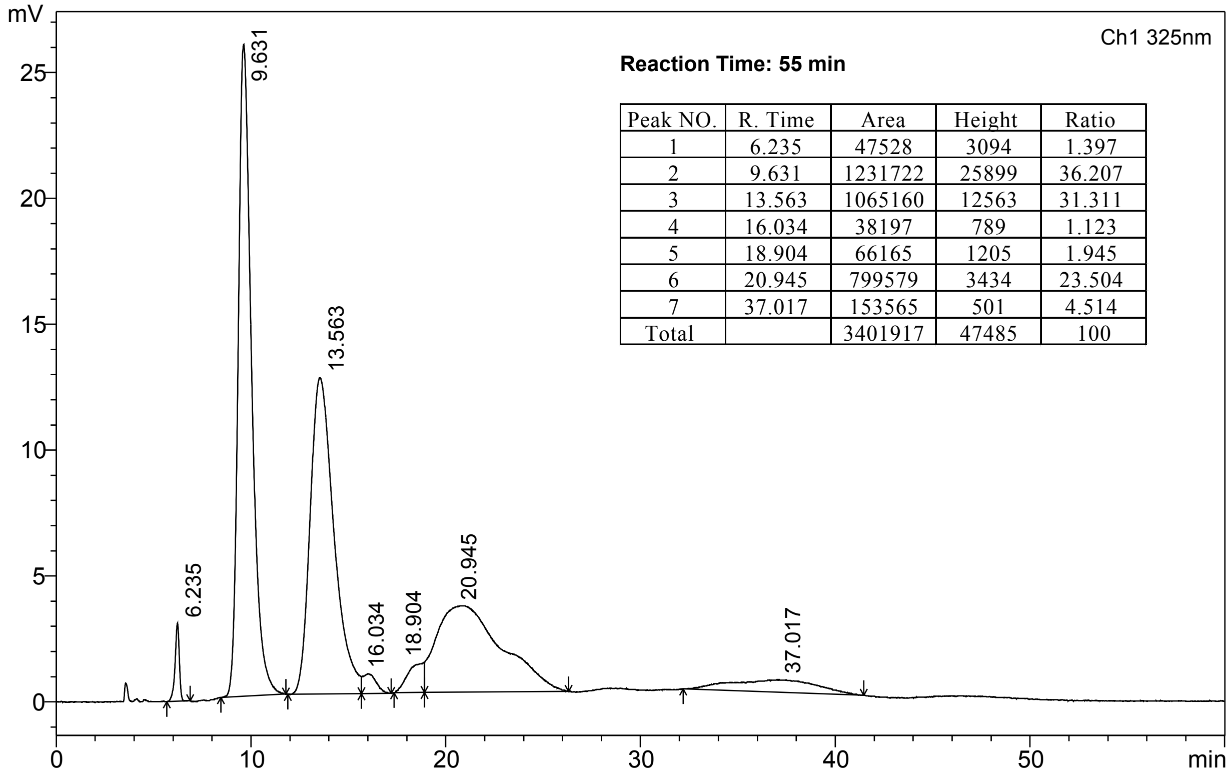

Peak NO.	R. Time	Area	Height	Ratio
1	6.235	47528	3094	1.397
2	9.631	1231722	25899	36.207
3	13.563	1065160	12563	31.311
4	16.034	38197	789	1.123
5	18.904	66165	1205	1.945
6	20.945	799579	3434	23.504
7	37.017	153565	501	4.514
Total		3401917	47485	100

Supplementary Figure 33. Time dependent HPLC spectra of the synthesis of **2** at 55 min.

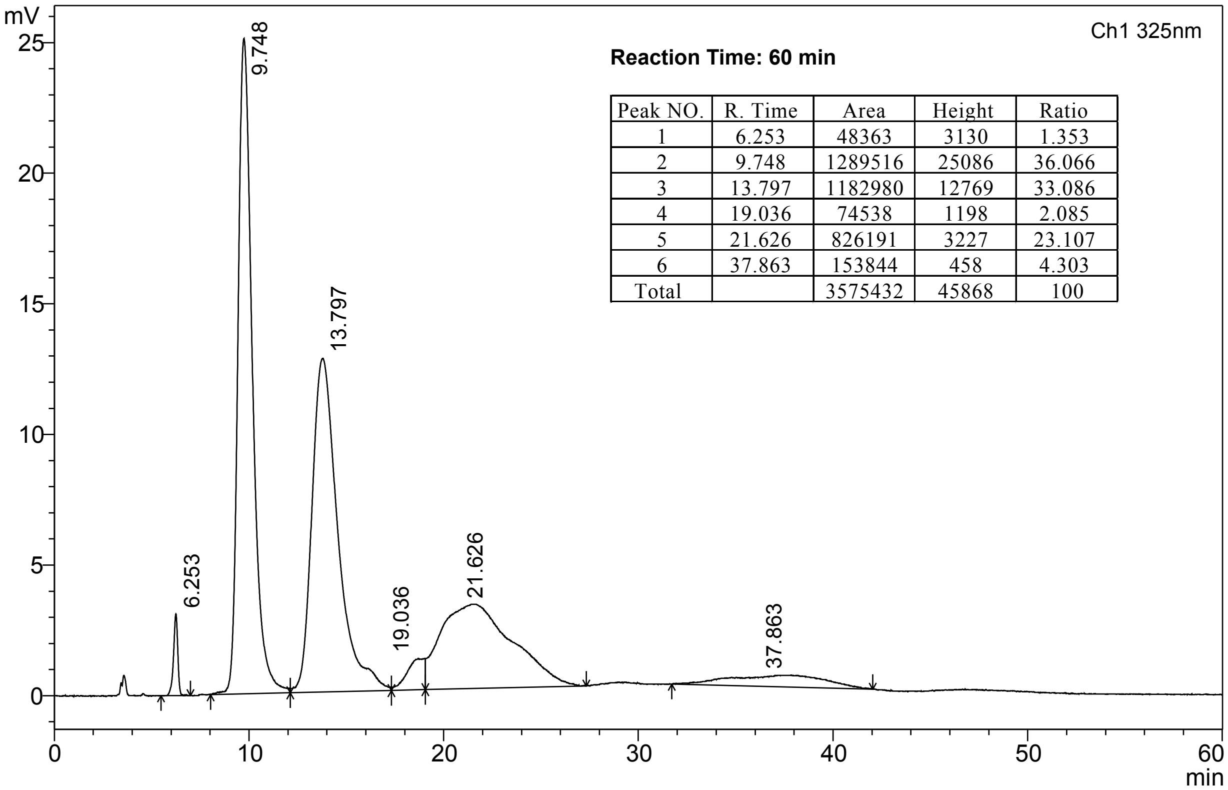

Peak NO.	R. Time	Area	Height	Ratio
1	6.253	48363	3130	1.353
2	9.748	1289516	25086	36.066
3	13.797	1182980	12769	33.086
4	19.036	74538	1198	2.085
5	21.626	826191	3227	23.107
6	37.863	153844	458	4.303
Total		3575432	45868	100

Supplementary Figure 34. Time dependent HPLC spectra of the synthesis of **2** at 60 min.

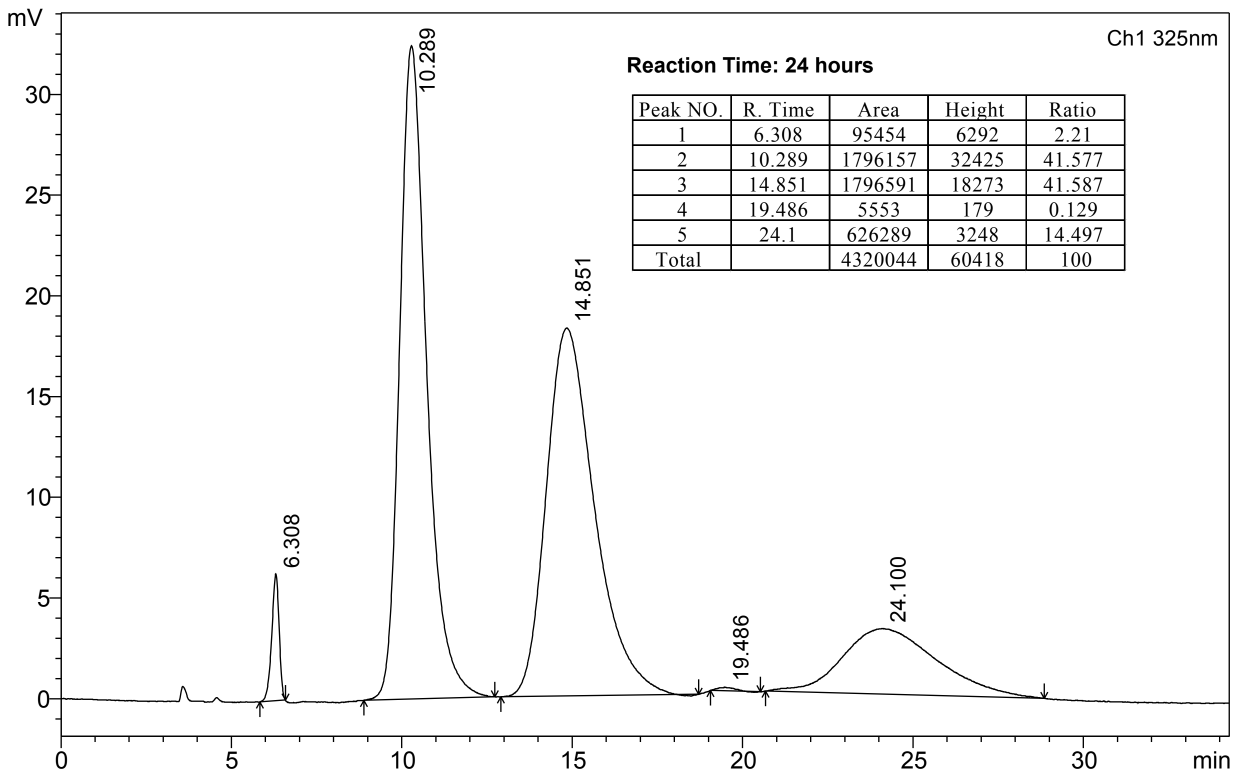

Peak NO.	R. Time	Area	Height	Ratio
1	6.308	95454	6292	2.21
2	10.289	1796157	32425	41.577
3	14.851	1796591	18273	41.587
4	19.486	5553	179	0.129
5	24.1	626289	3248	14.497
Total		4320044	60418	100

Supplementary Figure 35. Time dependent HPLC spectra of the synthesis of **2** at 24 hours.

Supplementary Table 1. Crystallographic data for FRP.

Compound	**1**	(*AAAA*)-**2**	(*CAAA*)-**2**	(*CCCC*)-**3**	(*CCCA*)-**3**
Empirical formula	$C_{228}H_{288}N_{12}$	$C_{252}H_{324}N_{12}$	$C_{252}H_{324}N_{12}$	$C_{252}H_{324}N_{12}$	$C_{252}H_{324}N_{12}$
Formula weight	3196.69	3521.21	3521.21	3521.21	3521.21
Temperature/K	100.01(10)	100.15	100.01(10)	100.01(10)	100.15
Crystal system	triclinic	trigonal	trigonal	trigonal	trigonal
Space group	P-1	R3	R3	R3	R3
a/Å	20.8082(4)	23.3017(3)	23.1134(9)	23.121(2)	23.2072(6)
b/Å	24.0721(4)	23.3017(3)	23.1134(9)	23.121(2)	23.2072(6)
c/Å	26.2995(4)	51.4329(7)	52.322(3)	51.666(2)	52.1786(11)
α/°	80.2288(13)	90	90	90	90
β/°	72.5101(15)	90	90	90	90
γ/°	75.3058(15)	120	120	120	120
Volume/Å^3	12090.9(4)	24184.9(7)	24207(2)	23921(4)	24337.1(13)
Z	2	3	3	3	3
ρ_{calc} /g cm^{-3}	0.878	0.725	0.725	0.733	0.721
μ/mm^{-1}	0.377	0.309	0.309	0.313	0.307
F(000)	3480	5760	5760	5760	5760
Crystal size/mm^3	0.4 × 0.4 × 0.4	0.5 × 0.4 × 0.3	0.5 × 0.4 × 0.4	0.3 × 0.15 × 0.15	0.4 × 0.3 × 0.25
Radiation	CuKα (λ = 1.54184)	CuKα (λ = 1.54178)	CuKα (λ = 1.54184)	CuKα (λ = 1.54184)	CuKα (λ = 1.54184)
2Θ range for data collection/°	6.548 to 131.474	7.588 to 147.58	7.65 to 131.322	7.648 to 136.43	7.618 to 122.332
Index ranges	$-24 \le h \le 24, -28 \le k \le 27, -30 \le l \le 28$	$-27 \le h \le 24, -22 \le k \le 28, -37 \le l \le 62$	$-25 \le h \le 24, -21 \le k \le 26, -60 \le l \le 54$	$-24 \le h \le 21, -20 \le k \le 27, -16 \le l \le 61$	$-22 \le h \le 26, -22 \le k \le 19, -60 \le l \le 38$
Reflections collected	80270	20428	29315	16509	16439
Independent reflections	40703 [R_{int} =0.0425]	14879 [R_{int} = 0.0388]	14704 [R_{int} = 0.0300]	10620 [R_{int} = 0.0675]	9834 [R_{int} = 0.0344]
Data/restraints/parameters	40703/349/2256	14879/195/794	14704/179/801	10620/228/792	9834/224/800
Goodness-of-fit on F^2	1.098	0.982	0.935	0.811	0.941
Final R indexes [I ≥ 2σ (I)]	R_1 = 0.0955, wR_2 = 0.2889	R_1 = 0.0960, wR_2 = 0.2428	R_1 = 0.0696, wR_2 = 0.1906	R_1 = 0.0725, wR_2 = 0.1663	R_1 = 0.0646, wR_2 = 0.1752
Final R indexes [all data]	R_1 = 0.1089, wR_2 = 0.3075	R_1 = 0.1069, wR_2 = 0.2551	R_1 = 0.0823, wR_2 = 0.2059	R_1 = 0.1038, wR_2 = 0.1850	R_1 = 0.0763, wR_2 = 0.1855
Largest diff. peak/hole / e Å^{-3}	0.83/-1.30	0.42/-0.29	0.21/-0.17	0.31/-0.23	0.20/-0.18
Flack parameter	none	0.2(9)	0.8(3)	-0.7(9)	-0.7(7)
CCDC#	1063965	1406534	1406540	1406657	1407221

Crystallographic data have been submitted to the Cambridge Crystallographic Database with according reference numbers and are available free of charge at http://www.ccdc.cam.ac.uk/data_request/cif.

Supplementary Methods

Synthesis of Tr-a: **Tr-a** was prepared according to the literature report[1]. Truxene (51.4 g, 150 mmol), dimethyl sulfoxide (500 mL), and potassium *t*-butoxide (101 g, 900 mmol) were mixed in a three necked round bottom flask (2 L) under nitrogen atmosphere. The flask was cooled to 0 ºC and stirred viciously. *n*-Butyl bromide (123 g, 900 mmol) was then slowly added to the flask, and the reaction was returned to room temperature. After 12 hours, other three batches of potassium *t*-butoxide (900 mmol × 3) and *n*-butyl bromide (900 mmol × 3) were added subsequently and stirred for 20 hours. The reaction was quenched with water (1 L) and ethyl acetate (300 mL) and the organic layer was extracted with ethyl acetate (1 L). The combined organic layer was washed with water (200 mL × 5), dried over magnesium sulfate, and filtered. After evaporation of solvents, the residue was passed through a pad of silica gel to give the final product as a white powder (yield 95%). Characterisation of **Tr-a** was reported by previous literature[2].

Synthesis of Tr-b: Tr-a (6.79 g, 10 mmol), chloroform (60 mL) and anhydrous $FeCl_3$ (20 mg) were added to a round bottom flask (100 mL) in nitrogen atmosphere and stirred until dissolved. The reaction was cooled to 0 ºC and bromine (2.5 mL) in chloroform (10 mL) was added slowly. After 24 hours, the reaction was quenched with saturated sodium thiosulfate aqueous solution (50 mL). The organic layer was washed with saturated sodium chloride solution and deionized water, and dried over anhydrous sodium sulfate. After evaporation of solvents, the solid was recrystallized from methanol to give **Tr-b** as a white powder (yield 99%). Characterisation of compound **Tr-b** was reported by previous literature[2].

Synthesis of TR: Tr-b (1.65 g, 1.8 mmol) and anhydrous ethyl ether (100 mL) were added to a flask under nitrogen. The flask was cooled to -78 °C and *n*-BuLi (6.6 mL, 15.8 mmol, 2.4 M in hexane) was slowly added and stirred until dissolved. After 30 min, the flask was gradually returned to room temperature and stirred for another 30 min. The reaction was cooled to -78 °C again and N,N-dimethylformamide (1.3 g, 17.6 mmol) was slowly added to the flask. The reaction was stirred overnight and quenched with aqueous hydrochloric acid (2 M, 100 mL). The organic layer was washed with aqueous sodium chloride solution (20 mL × 3) and dried over sodium sulfate. The solvent was evaporated under vacuum and the solid was purified by column chromatography with hexane:dichloromethane = 9:1 (v/v) to give **TR** as a white powder (yield, 70%)[3].

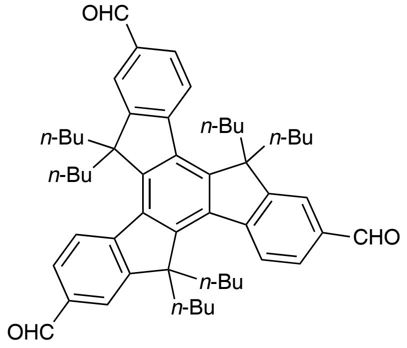

TR

TR. ^{1}H NMR (500 MHz, $CDCl_3$, ppm, δ): 10.16 (s, 3H), 8.57 (d, J = 8.20 Hz, 3H), 8.03 (d, J = 1.44 Hz, 3H), 7.79 (dd, J_1 = 8.16 Hz, J_2 = 1.48 Hz, 3H), 2.94–3.03 (m, 6H), 2.19–2.29 (m, 6H), 0.80–1.00 (m, 12H), 0.47–0.58 (m, 6H), 0.36–0.47 (m, 24H); ^{13}C NMR (126 MHz, $CDCl_3$): δ192.23, 154.42, 149.20, 145.69, 138.09, 135.08, 129.70, 125.10, 122.50, 62.09, 56.22, 36.69, 26.64, 22.80, 13.83. HRMS (*m/z*): $[M+Na]^+$ calcd for $C_{54}H_{66}O_3Na$, 785.49041; found, 785.49156. analysis (% calcd, % found for $C_{54}H_{66}O_3$): C (84.99, 84.72), H (8.72, 8.82), O (6.29, 6.46).

Synthesis of 1. TR (400 mg, 0.5246 mmol), ethylenediamine (47.2 mg, 0.7869 mmol), and trifluoroacetic acid (4.485 mg, 0.0393 mmol) were reacted in toluene (400 mL) at room temperature following the general procedure of synthesizing octahedra. Product: light yellow solid, yield 95%.

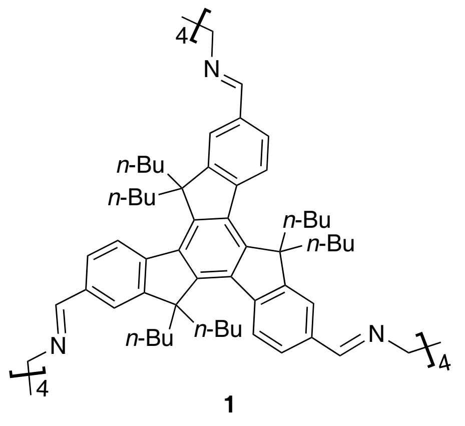

1

1. ^{1}H NMR (500 MHz, CD_2Cl_2, ppm, δ): 8.40 (s, 12H), 8.15 (d, J = 8.25 Hz, 12H), 7.84 (s, 12H), 7.38 (d, J = 7.79 Hz, 12H), 4.27 (d, J = 8.64 Hz, 12H), 3.96 (d, J = 9.02 Hz, 12H), 2.76–2.89 (m, 12H), 2.42–2.56 (m, 12H), 1.96–2.08 (m, 12H), 1.86−1.96 (m, 12H), 0.68–0.89 (m, 24H), 0.30–0.43 (m, 48H), 0.17–0.29 (m, 36H), 0.08–0.16 (m, 12H), (-0.36)–(-0.15) (m, 48H). ^{13}C NMR (125 MHz, CD_2Cl_2): δ 162.33, 154.24, 146.71, 142.66, 137.98, 134.86, 128.98, 124.78, 119.36, 62.36, 55.83, 36.62, 36.21, 26.95, 25.33, 23.21, 21.96, 13.99, 12.86. HRMS (*m/z*): $[M+2H]^{2+}$ calcd for $C_{228}H_{290}N_{12}$,

1599.15581; found, 1599.15475.

Synthesis of 2. TR (400 mg, 0.5246 mmol), (R,R)-cyclohexane-1,2-diamine (89.9 mg, 0.7869 mmol), and trifluoroacetic acid (4.485 mg, 0.0393 mmol) were reacted in toluene (400 mL) at room temperature following the general procedure of synthesizing octahedra. The kinetic product: yellow solid, 98%. Ratio of (*AAAA*)-**2**, (*CAAA*)-**2** and (*CCAA*)-**2** in the kinetic product was determined by chiral HPLC to be 42:42:14. The reaction was then heated up to 110 ºC in a sealed tube for 48 hours to give the thermodynamic product (*AAAA*)-**2**.

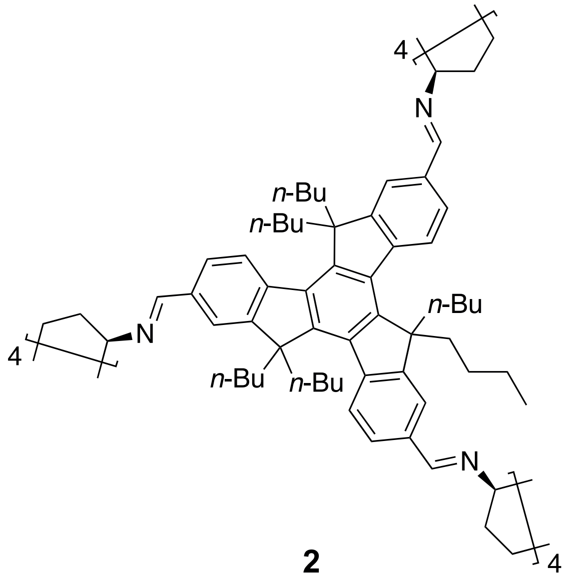

2

2. ^{1}H NMR (500 MHz, CD_2Cl_2, ppm, δ): 8.38 (s, 12H), 8.16 (d, J = 8.17 Hz, 12H), 7.78 (s, 12H), 7.30 (d, J = 8.02 Hz, 12H), 3.46–3.55 (m, 12H), 2.79–2.88 (m, 12H), 2.38–2.49 (m, 12H), 1.96–2.05 (m, 12H), 1.87–1.95 (m, 24H), 0.67–0.89 (m, 24H), 0.28−0.40 (m, 48H), 0.08–0.28 (m, 48H), (–0.32)–(–0.20) (m, 48H). ^{13}C NMR (125 MHz, CD_2Cl_2): δ 160.39, 154.32, 146.63, 142.56, 138.22, 135.11, 129.67, 124.75, 118.56, 76.00, 55.84, 36.68, 36.08, 33.71, 26.95, 25.22, 25.09, 23.23, 21.89, 14.00, 12.86. HRMS (*m/z*): $[M+3H]^{3+}$ calcd for $C_{252}H_{327}N_{12}$, 1174.5288; found, 1174.5042.

Single-crystal X-ray diffraction. FRP were dissolved in toluene (1 mL) and layered with hexane (2 mL) on top. Transparent block crystals were grown within three to seven days. Single crystal X-ray diffraction data were collected on Rigaku SuperNova X-Ray single crystal diffractometer using Cu Kα (λ = 1.54184 Å) micro-focus X-ray sources. Suitable crystal was collected, covered with protective oil and mounted on X-ray diffractometer. The octahedra contain only light elements (i.e., C, H, and N) and the crystal cracks within seconds in air. The crystal was kept in 100 K with liquid nitrogen stream during the unit cell determination and full data collection. The raw data were collected and reduced by CrysAlisPro software, the structures were solved with the SHELXS[4] or SHELXT[4] using Direct Methods and refined with the SHELXL[4] using CGLS minimization, and OLEX2[5] were used as GUI.

Refinement details: For each crystal of **1**, (*AAAA*)-**2**, (*CAAA*)-**2**, (*CCCC*)-**3**, and (*CCCA*)-**3**, all non-hydrogen atoms were refined anisotropically. Hydrogen atoms were placed at calculated positions using the riding model and refined isotropically. The instructions AFIX 23 and AFIX 43 were used for the hydrogen atoms on the secondary $-CH_2-$ and the aromatic C-H, respectively, with the parameter of U_{iso}=1.2 U_{eq}. The instruction AFIX 33 was used for the hydrogen atoms on the highly disordered terminal $-CH_3$ groups with the parameter of U_{iso}=1.5 U_{eq}. No Shelx restraint was applied to the skeleton of the octahedra, i.e. truxene faces and diamine vertices. Nevertheless, the flexible butyl groups are expected to be highly disordered, as they are flexible and vibrate randomly in the large voids in the crystal. Therefore, necessary Shelx restraints (i.e., DELU, SIMU, and EADP) were applied to the butyl groups to result in a reasonable model. Specifically, the anisotropic displacement parameters of disordered atoms in butyl groups were restrained to be equal within an effective standard deviation of 0.001 using the DELU command. U_{ij} values of disordered atoms of butyl groups were constrained to be similar using the SIMU command. Atomic displacement parameters (ADPs) of different parts of disordered atoms were restrained using the EADP command. There are large voids between the octahedra in crystal, filling with highly disordered solvent molecules. A satisfactory disorder model for the solvent molecules was not found, therefore the OLEX2 Solvent Mask routine (similar to PLATON/SQUEEZE) was used to mask out the disordered density.

Note that, for each chiral octahedra (*AAAA*)-**2**, (*CAAA*)-**2**, (*CCCC*)-**3**, and (*CCCA*)-**3**, the absolute configuration in crystal was assigned by reference to an unchanging chiral centre in the assembly procedure, i.e., the chiral carbon atoms of the reagent (1*R*,2*R*)-diaminocyclohexane or (1*S*,2*S*)-diaminocyclohexane, instead of anomalous dispersion effects in diffraction measurements on the crystal and the derived Flack x parameter. The octahedron crystal contains only light atoms (N, O, C, H) and has large void regions, therefore it is extremely difficult to determine the absolute configuration by the Flack x parameter with such a large uncertainty. Instead, the diaminocyclohexane reagents used in the assembly

procedure have known absolute configurations, and (1*R*,2*R*)-diaminocyclohexane or (1*S*,2*S*)-diaminocyclohexane does not change its absolute configuration during the assembly process. Therefore, (1*R*,2*R*)-diaminocyclohexane or (1*S*,2*S*)-diaminocyclohexane fragments can be used as an internal reference to determine the absolute configurations of the octahedra (*AAAA*)-**2**, (*CAAA*)-**2**, (*CCCC*)-**3**, and (*CCCA*)-**3**.

Kinetics of the Synthesis. TR (122 mg, 0.160 mmol), (R,R)-cyclohexane-1,2-diamine (14.4 mg, 0.240 mmol) and trifluoroacetic acid (1.37 mg, 0.0120 mmol) were reacted in toluene (100 mL) at 298 K following the general procedure of synthesizing the octahedra. At certain time intervals, the reaction solution (0.2 mL) were drawn out and injected into the mixed solution of hexane:ethanol (v/v) = 75:25 (1.4 ml). The diluted solutions were immediately refrigerated in liquid nitrogen for the latter chiral-HPLC, MS, UV-Vis, and CD characterisations. HPLC measurements were carried out in the mobile phase of hexane:ethanol = 75:25 (v/v) with 0.1% diethylamine . The spectra of refrigerated solutions did not change after being kept in liquid nitrogen for more than 24 hours.

Computational methods. Structures were constructed on the basis of the crystal structures and adjusted to follow the symmetries of *T* (*AAAA* and *CCCC*), C_3 (*CAAA* and *CCCA*) and C_2 (*CCAA*). All the structural optimization and energy calculation were carried out in Materials Studios 7.0 as a licensed product of BIOVIA. Electrostatic potential energy between atoms arises from partial charges for each atom assigned by the COMPASS II force field[6,7]. Gasteiger charges and the smart algorithm were used within ultrafine calculation quality (2.0×10^{-5} kcal mol^{-1} energy, 0.001 kcal mol^{-1} Å^{-1} Force, 1.0×10^{-5} Å displacement with 5000 iterations for convergence tolerance). Coordinates of all optimized structures are provided in Supplementary Excel file.

The CD spectra were calculated at ZINDO[8,9] semi-empirical level with Gaussian 09[10] and used the energy-minimized structures optimized by the COMPASS II force-field. The electronic transitions were then fitted to a Lorentzian distribution to generate the spectra.

Supplementary Reference

1 Tsuji, H. *et al.* Tripyridyltruxenes: Thermally stable cathode buffer materials for organic thin-film solar cells. *Asian J. Org. Chem.* **1**, 34–37 (2012).
2 Zhou, H. *et al.* Synthesis of star-shaped monodisperse oligo(9,9-di-n-octylfluorene-2,7-vinylene)s functionalized truxenes with two-photon absorption properties. *Org. Biomol. Chem.* **9**, 1600–1607 (2011).
3 Yuan, S., Chen, H., Zhang, Y. & Pei, J. Rigid linear and star-shaped π-conjugated 2,2':6',2''-terpyridine ligands with blue emission. *Org. Lett.* **8**, 5701–5704 (2006).
4 Sheldrick, G. Crystal structure refinement with SHELXL. *Acta Cryst. Sect. C* **71**, 3-8 (2015).
5 Dolomanov, O. V., Bourhis, L. J., Gildea, R. J., Howard, J. A. K. & Puschmann, H. OLEX2: a complete structure solution, refinement and analysis program. *J. Appl. Crystallogr.* **42**, 339–341 (2009).
6 Sun, H. COMPASS: An ab initio force-field optimized for condensed-phase applications — overview with details on alkane and benzene compounds. *J. Phys. Chem.*, 7338–7364 (1998).
7 Sun, H. & Ren, P. The compass force field: parameterization and validation for phosphazenes. *Comput. Theor. Polym. Sci.*, 229–246 (1998).
8 Telfer, S. G., Tajima, N. & Kuroda, R. CD spectra of polynuclear complexes of diimine ligands: Theoretical and experimental evidence for the importance of internuclear exciton coupling. *J. Am. Chem. Soc.* **126**, 1408–1418 (2004).
9 Telfer, S. G., Tajima, N., Kuroda, R., Cantuel, M. & Piguet, C. CD spectra of d-f heterobimetallic helicates with segmental di-imine ligands. *Inorg. Chem.* **43**, 5302–5310 (2004).
10 Frisch, M. J. *et al.*, Gaussian 09, revision. B.01 (2010).
